# Are reasons for first using cannabis associated with subsequent cannabis consumption (standard THC units) and psychopathology?

**DOI:** 10.1136/bmjment-2025-301810

**Published:** 2025-08-27

**Authors:** Edoardo Spinazzola, Hannah Degen, Isabelle Austin-Zimmerman, Giulia Trotta, Edward Chesney, Zhikun Li, Luis Alameda, Bok Man Leung, Yifei Lang, Andrea Quattrone, Diego Quattrone, Erika Castrignanò, Kim Wolff, Robin Murray, Tom P Freeman, Marta Di Forti

**Affiliations:** 1 Psychosis Studies, King’s College London, London, UK; 2 South London and Maudsley NHS Foundation Trust, London, UK; 3 Psychosis Studies, King’s College London Institute of Psychiatry, Psychology and Neuroscience, London, UK; 4 Social, Genetic, and Developmental Psychiatry Centre, King’s College London Institute of Psychiatry Psychology and Neuroscience, London, UK; 5 King’s College London Department of Addictions, London, UK; 6 South London and Maudsley Mental Health NHS Trust, London, UK; 7 Department of Psychiatry, The University of Hong Kong Li Ka Shing Faculty of Medicine, Hong Kong, Hong Kong; 8 University of Porto Institute of Biomedical Sciences Abel Salazar, Porto, Portugal; 9 King’s College London Department of Forensic and Analytical Science, London, UK; 10 Psychology, University of Bath, Bath, UK

**Keywords:** Adult psychiatry, Cross-Sectional Studies, Substance misuse, Substance-Related Disorders, Marijuana Abuse

## Abstract

**Background:**

Reasons for first using cannabis (RFUC) may influence later use patterns and mental health outcomes. However, limited research has explored self-medication versus social RFUCs in depth, and their associations with cannabis use patterns and psychopathology in the general population.

**Objectives:**

We examined RFUCs and their associations with (1) reasons for continuing cannabis use, (2) weekly THC (delta-9-tetrahydrocannabinol) unit consumption and (3) symptoms of paranoia, anxiety and depressive symptoms.

**Methods:**

We analysed data from the Cannabis&Me (CAMe) population survey (March 2022–July 2024), including 2573 (75.9%) current and 816 (24.1%) past cannabis users aged 18 years or older.

**Findings:**

Participants reported a mean weekly consumption of 206 THC units (SD=268). Initiating cannabis use for anxiety (β=36.22, p=3.3e−03), depression (β=40.37, p=1.74e−03) or because ‘family members were using it’ (β=87.43, p=1.22e−09) was associated with higher weekly THC units. RFUC to relieve physical discomfort (β=8.89, p=4.12e−07), pain (β=7.24, p=5.56e−06), anxiety (β=9.67, p=1.63e−16), depression (β=9.12, p=1.21e−13) and minor psychotic symptoms (β=16.46, p=1.2e−04) were linked to higher paranoia scores. Similar associations were observed for anxiety and depression. Conversely, starting for fun (β=−3.71, p=3.49e−05) or curiosity (β=−2.61, p=5e−03) was associated with lower paranoia and anxiety. RFUC for ‘boredom’ was linked to increased depression (β=1.09, p=3.8e−03).

**Conclusions:**

Initiating cannabis use for self-medication is associated with higher average THC consumption, and increased anxiety, depression and paranoia.

**Clinical implications:**

Asking individuals why they first used cannabis may serve as a cost-effective screening tool to identify those who could benefit from monitoring, support, or referral to intervention services.

**WHAT IS ALREADY KNOWN ON THIS TOPIC**

- Motivations for cannabis use are associated with frequency of use and increased probability of developing clinical psychosis.

**WHAT THIS STUDY ADDS**

- We analysed data from 3389 people with lifetime cannabis use, between March 2022 and July 2024, from the Cannabis&Me study, the largest independent study of its kind.
- We applied and validated the weekly THC (delta-9-tetrahydrocannabinol) units as a standardised measure of consumption.
- First evidence that starting to use cannabis to reduce either physical or psychological discomfort, separating the two groups usually analysed together, is associated with (1) high weekly THC unit consumption and (2) higher paranoia, anxiety and depressive symptoms.

**HOW THIS STUDY MIGHT AFFECT RESEARCH, PRACTICE OR POLICY**

- Specific subgroups of people who use cannabis appear to be at greater risk of developing adverse outcomes and therefore may require targeted treatment interventions.
- Given the ongoing trend towards legalisation of cannabis for both recreational and medicinal purposes, particular attention should be given to those who report self-medicating from either psychological or physical distress.
- Asking people why they started using cannabis could become an easy and cheap screening tool for identifying cannabis users who require monitoring, support and triage to interventions.

## Background

 The reason why an individual starts and continues to use cannabis may predict subsequent patterns and mental health outcomes.^1 2^ In a recent case–control study, we found that both people with first-episode psychosis and healthy controls were more likely to start using cannabis with friends (75.6%) than for any other reason,^3^ confirming previous evidence that most people use cannabis in a social context and for hedonistic reasons.^1 4^ Our path analysis suggested that those who start using cannabis ‘to feel better’ were significantly more likely to progress to daily use of high-potency cannabis and to suffer a first episode of psychosis later.^3^ Heavy cannabis use is associated with various adverse health outcomes, such as psychosis,^5^ anxiety^6^ and major depression.^7^ However, only a subset of people who use cannabis experience such adverse effects.^8^ Early onset and frequent consumption of high-potency cannabis appear to be linked to such outcomes.^9^

Paranoia, a common adverse effect of heavy cannabis use, involves irrational fears of others’ harmful intentions and has been widely studied in experimental^10^ and epidemiological studies.^11^ While a symptom of psychosis, paranoia also exists in the general population, affecting health, emotional well-being, social functioning and social inclusion.^12^ The Green *et al* Paranoid Thoughts Scale (GPTS) was built to measure such trait paranoid ideation in the general population.^13^

To investigate whether reasons for first using cannabis (RFUC) can be used to identify those cannabis users more likely to endorse harmful patterns at an early stage, we sought to estimate associations between RFUC, subsequent cannabis use and mental health symptoms in a general population sample. London was identified as a key global arena for targeted intervention, given the high levels of cannabis use regionally and the association with elevated incidence of psychotic disorders.^14^ To overcome a lack of standardisation in cannabis research, cannabis use was assessed using a direct measure of the primary psychoactive constituent, ‘the standard THC unit’, as recommended by the US National Institutes of Health.^15^

### Objectives

Using data from a large population sample of cannabis users (Cannabis&Me (CAMe) project), who have never been diagnosed or treated with a psychotic disorder, we sought to (1) identify the most common RFUCs, (2) investigate the correlation between RFUC and reasons for continuing to use cannabis (RCUC) and for the first time, (3) to determine which RFUCs are associated with a standardised measure of cannabis use (higher THC units, delta-9-tetrahydrocannabinol, consumed) which takes into account both the frequency and the potency of the cannabis consumed, and (4) explore how RFUCs are linked to higher levels of anxiety, depression and paranoia.

## Methods

### Study design

We used data from the CAMe study online survey, a population-based, non-clinical sample of adult current, past (not in the last year) and never cannabis users.

### Sample

The analyses in this paper are based on data collected between 30 March 2022 and 31 July 2024, comprising a total sample of N=3389 participants with cannabis use. To achieve a sample as representative as possible of the London general population, multiple channels, major social media platforms, targeted efforts and collaboration with marketing agency experts in sample recruitment (https://literalhumans.com/) were used.

Participants were aged≥18 years, residents of the London area or able to travel, English speakers and consented to be contacted for a potential follow-up face-to-face assessment. Those with a current or past diagnosis of psychotic disorders were excluded. A subsample of N=88 participants took part in a subsequent face-to-face assessment. This included a blood sample collection with measurement of THC and its metabolites.

### Measures and assessments

Participants completed a 40-min online survey through the http://www.onlinesurveys.ac.uk/ platform. Socio-demographic data were collected using the Medical Research Council Sociodemographic Schedule.^16^ Participants were assessed using the Generalised Anxiety Disorder Scale (GAD-7),^17^ the Patient Health Questionnaire (PHQ-9)^18^ and trait paranoid ideation was assessed with the GPTS.^13^ For all our cannabis use measures, we used a modified version of the Cannabis Experiences Questionnaire.^19^ The changes to the questionnaire included expanding the RFUC response options from 4 to 10, enabling us to capture a broader range of RFUC. This approach aimed to maximise coverage by reflecting the widest possible variety of motivations while also distinguishing key domains, such as (1) self-medication/coping and (2) enhancement or social use (see also online supplemental file 1).

Participants were asked, ‘Why did you first try cannabis?’, with multiple-choice responses: (1) my friends were using it; (2) my family members were using it; (3) to feel better (to get relief from physical discomfort); (4) to feel better (to get relief from pain); (5) to feel better (to get relief from anxiety symptoms); (6) to feel better (to get relief from depressive symptoms); (7) to feel better (to get relief from experiences such as hearing voices or feeling suspicious); (8) for curiosity; (9) for fun; (10) to overcome boredom. The following abbreviations are used throughout this paper: (1) ‘Friends’, (2) ‘Family’, (3) ‘Better—physical discomfort’, (4) ‘Better—pain’, (5) ‘Better—anxiety’, (6) ‘Better—depression’, (7) ‘Better—psychosis’, (8) ‘Curiosity’, (9) ‘Fun’ and (10) ‘Boredom’. Subjects were able to provide up to 10 RFUC. In addition, participants were asked about their RCUC: ‘Why do you continue to use cannabis?’, and they could select one of the following responses: (1) I like the effect, it gives me a buzz; (2) It makes me feel relaxed; (3) It makes me feel less nervous and anxious; (4) it makes me feel more sociable; (5) other. The following abbreviations were used: (1) ‘buzz’, (2) ‘relaxed’, (3) ‘less nervous and anxious’, (4) ‘sociable’ and (5) ‘other’.

Frequency of cannabis use was categorised as never or occasional use=0; monthly or less than monthly=1; weekly or less=2; more than once a week=3; daily=4.

Self-reported data on the frequency and potency of the cannabis used were collected, as demonstrated to be reliable measures of the quantity of THC used.^19^,^21^ Participants could report directly the potency (THC%) of the cannabis used or the name of the type used; information on the quantity of cannabis used g/week was also collected (see online supplemental file 1). THC consumption was estimated using standard weekly THC units (1 unit=5 mg THC), consistent with the National Institutes of Health mandating reporting.^15^ Standard THC units provide a direct measure of the primary psychoactive constituent in cannabis, THC. To reduce the variability of standard THC unit estimates, winsorisation was applied.^22^ Quantitative analysis using liquid chromatography coupled with mass spectrometry measured THC and its metabolites.

Several strategies were applied to ensure data completeness (see online supplemental file 1).

### Statistical analyses

Statistical analyses were performed using R Studio (V.4.2.1). Descriptive statistics summarised the sample, with continuous variables expressed as means and SD and categorical variables presented as frequencies. Group differences were evaluated using t-tests or Mann-Whitney U tests (online supplemental table S3; table 1). Since participants could provide more than one RFUC, the number of reported RFUC and the overlap (online supplemental figure S3) were calculated, as well as the subsample with current cannabis use, followed by a test for the correlation between RFUC and RCUC (online supplemental figure S4).

**Table 1:** Participants’ characteristics in the working sample (N=3.389 past and current cannabis users)

Variable	Statistics	Descriptor
Demographics		
Age	M (SD), median (IQR)	30.9 (9.8), 29 (12)
Sex	Males (%)	2103 (62.1)
Ethnicity	White/white other (%)	2206 (65.1)
Employment status	Employed (%)	2802 (85.4)
Years of education	M (SD), median (IQR)	15.9 (3.9), 16 (4)
Cannabis use		
Cannabis use	Yes (%)	3389 (100)
Cannabis use	Current use (%)	2573 (75.9)
Cannabis use	Past use only (%)	816 (24.1)
Frequency of use	Daily use (%)	1719 (50.9)
THC unit	M (SD), median (IQR)	206 (268), 112 (232)
Age first tried	M (SD), median (IQR)	16.7 (5.6), 16 (4)
Psychopathology		
GAD-7	M (SD), median (IQR)	6.1 (5.3), 5 (7)
PHQ-9	M (SD), median (IQR)	7.8 (6.5), 6 (9)
GPTS-A	M (SD), median (IQR)	27.4 (11.9), 23 (15)
GPTS-B	M (SD), median (IQR)	23.3 (11.9), 17 (9)
GPTS-TOT	M (SD), median (IQR)	50.8 (22.5), 42 (24)

GAD-7, Generalised Anxiety Disorder assessment; GPTS, Green *et al* Paranoid Thoughts Scale; GPTS-A, ideas of reference; GPTS-B, persecution; GPTS-TOT, GPTS total score; M, mean; PHQ-9, Patient Health Questionnaire for depression.

A series of adjusted linear regressions were carried out to test the association between the following 10 RFUC, age at first cannabis use, level of cannabis use in standard THC units, GPTS^TOTAL^, GAD and PHQ-9 scores. The Bonferroni correction was applied for five independent tests (p<0.01). Missing data (25.3%) for models that included the standard THC unit were mainly due to ambiguous free-test entries for g/week. The Multiple Imputation by Chained Equations (MICE, in R software) was used to address non-random missingness.^23 24^

Predictive Mean Matching was applied for continuous variables to ensure realistic imputations. Five imputations (m=5) and a maximum of five iterations (maxit=5) were used to ensure result convergence and stability. All analyses were adjusted for age, sex, ethnicity, education, and employment status.

To ensure additional robustness, sensitivity analysis using a series of multinomial logistic regressions adjusted for age, sex, ethnicity, years of education and employment status was used to test the association between each of the RFUC and a composite measure of the frequency of cannabis use (online supplemental table S8).

### Validation of the standard THC unit measure

Blood THC concentrations, from a subgroup who participated in a later face-to-face assessment, were strongly correlated with weekly standard THC units (Spearman’s r=0.77, p=3.08e−13) as well as frequency of use (Spearman’s r=0.63, p=1.73e−09) (see online supplemental paragraph 2.9).

## Findings

### Sociodemographic characteristics

The characteristics of the sample population of 3389 participants in terms of general sociodemographics, measures of cannabis use and psychopathology (GPTS^TOTAL^, GAD and PHQ-9) are summarised in table 1. Data from respondents who reported past cannabis use 816 (24.1%) or who reported current cannabis use 2573 (75.9%) were considered (online supplemental figure S1, recruitment flow chart).

Participants had a mean age of 30.9 years (SD: 9.8); 2103 (62.1%) were male, 2206 (65.1%) were either white British or any other white group, 427 (12.6%) reported mixed ethnicity, 380 (11.2%) black British ethnicity and 376 (11.1%) Asian or British Asian ethnicity. The majority were employed, 2802 (85.4%) and reported a mean of 15.9 years of education (SD: 3.9). The average age at first cannabis use was 16.7 years (SD: 5.6), and half (1719, 50.9%) of the sample used cannabis daily (table 1). The mean weekly consumption of standard THC units was 206 (SD: 268). In the subsample of 88 participants with available THC blood level data, the mean concentration was 11.5 ng/mL (SD: 10.8).

More than two-thirds of participants (69.8%, 2366) reported starting to use cannabis because of ‘friends’; while 62.3% (2111) stated starting for ‘curiosity’ and 52.7% (1787) for ‘fun’. Much lower numbers reported starting use for ‘Better—anxiety’ (15.4%, 521); ‘Better—depression’ (13.8%, 469); to reduce ‘boredom’ (11.8%, 399); because of ‘family’ members (10.5%, 355); ‘Better—pain’ (7.6%, 257); ‘Better—physical discomfort’ (6%, 204). Online supplemental figure S3 reports overlapping answers for the RFUC variables.

### RCUC and correlation between RFUC and RCUC

**Figure 1:**
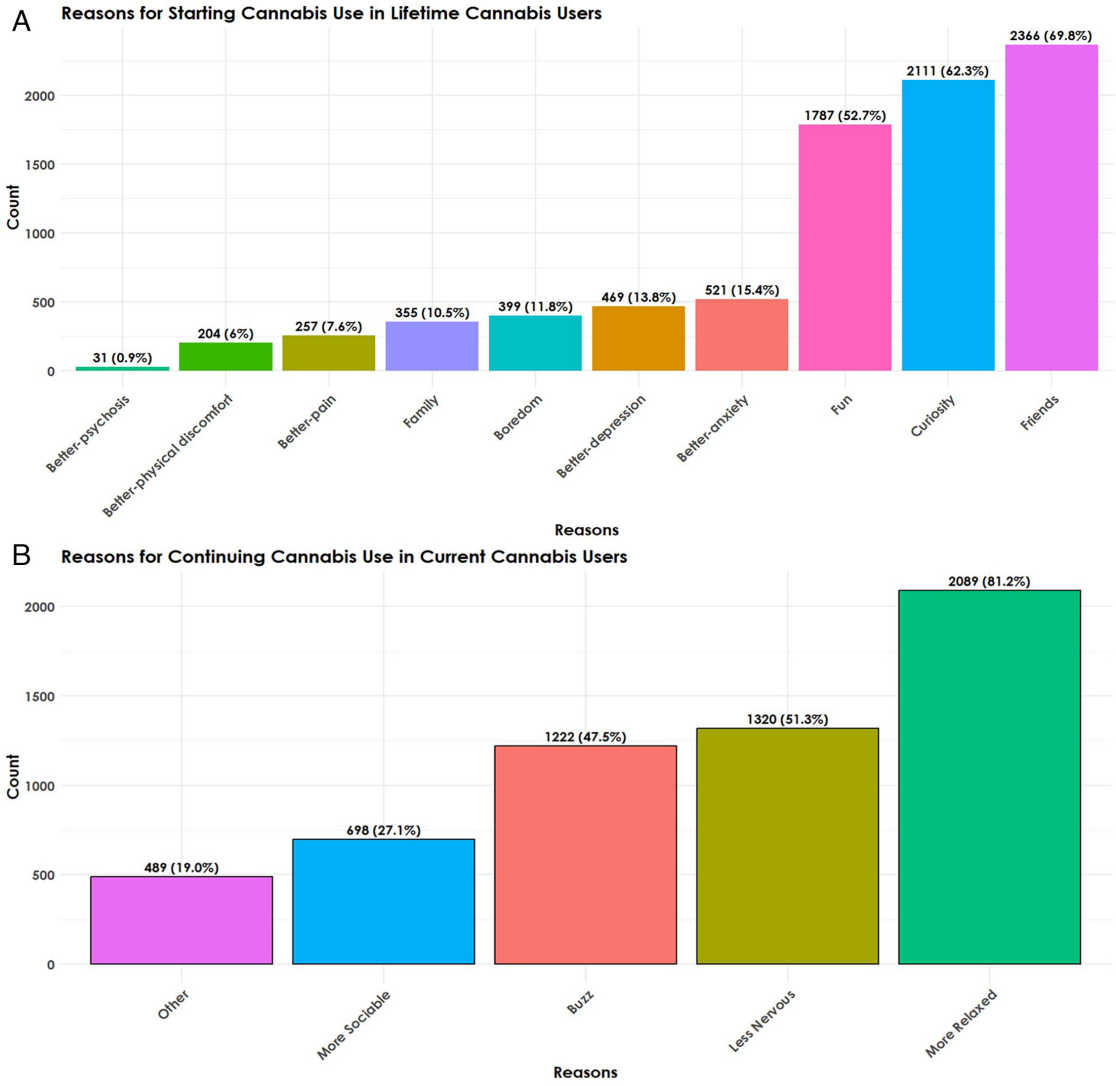
(**A**) Reasons for first using cannabis as depicted by the number of participants and percentage of sample with lifetime cannabis use. (**B**) Reasons for continuing to use cannabis as depicted by the number of people and percentage of sample with current cannabis use.

The RCUC distribution for those answering, ‘Why do you continue to use cannabis?’ (N=2573) was calculated as a subset with 81.2% (2,089) reporting ‘relaxed’ as their RCUC. About half (51.3%, 1320) reported RCUC ‘nervous’, ‘buzz’ (47.5%, 1222), while less than one-third (27.1%, 698) reported ‘sociable’, or any ‘other’ reasons (19%, 489) (figure 1).

There was a moderate positive correlation between RFUC for ‘fun’ and RCUC to feel ‘relaxed’ (r=0.41), and between RFUC with ‘friends’ and RCUC ‘relaxed’ (r=0.39) among current cannabis users. We also found a low-to-moderate correlation between RFUC ‘Better—anxiety’ and RCUC ‘nervous’ (r=0.36), between RFUC ‘curiosity’ and RCUC ‘relaxed’ (r=0.38), and between ‘fun’ and RCUC ‘buzz’ (r=0.37). RFUC ‘Better—pain’ showed weak but still significant levels of correlation with RFUC ‘nervous’ (r=0.19), ‘sociable’ (r=0.15) and ‘relaxed’ (r=0.14). Similarly, RFUC ‘Better—physical discomfort’ was weakly but significantly correlated with RCUC ‘nervous’ (r=0.16), ‘relaxed’ (r=0.13) and ‘sociable’ (r=0.12) (online supplemental figure S4).

### Associations between age at first cannabis use and RFUC

**Figure 2:**
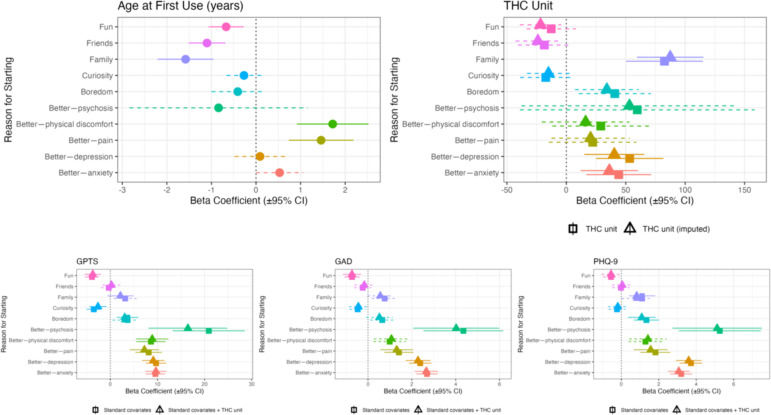
Adjusted linear regressions to test the associations between RFUC and (1) age at first cannabis use, (2) THC unit, (3) GPTS-tot, (4) GAD and (5) PHQ-9. All the analyses were adjusted for sex, age, ethnicity, employment status and years of education. Additional adjustment for THC is also reported. We applied a Bonferroni correction for 10 independent reasons for first using cannabis, setting the significance threshold at p<0.005. Significant p values (below this threshold) are shown in solid lines, while non-significant p values, following correction, are represented with dotted lines. GAD, Generalised Anxiety Disorder assessment; GPTS, Green *et al* Paranoid Thoughts Scale; GPTS-tot, Green *et al* Paranoid Thoughts Scale Total Score; PHQ-9, Patient Health Questionnaire for depression; RFUC, reasons for first using cannabis.

A series of linear regressions adjusted for age, sex, ethnicity, employment status and years of education indicated that RFUC with ‘friends’ (β=−1.1; (95% CI: −1.52 to –0.68), p=3.17e−07), with ‘family’ (β=−1.58, (95% CI: −2.21 to –0.95), p=9.5e−07) and for ‘fun’ (β=−0.67; (95% CI: −1.05 to –0.28), p=6.57e−04), were associated with lower age of first cannabis use, while RFUC ‘Better—physical discomfort’ (β=1.72, (95% CI: 0.91 to 2.52), p=2.87e−05) and ‘Better—pain’ (β=1.46, (95% CI: 0.73 to 2.19), p=9.42e−05) were associated with age of onset of use (figure 2; online supplemental table S6).

### Association between RFUC and standard THC unit

Linear regressions tested the association between each of the RFUC and THC exposures measured in weekly standard THC units. RFUC ‘family’ (THC mean: 286.9 (SD: 357); β=87.4 (95% CI 59.4 to 115.5), p=1.22e−09), ‘Better—anxiety’ (THC mean: 248 (SD: 294.1); β=36.2 (95% CI 12.1 to 60.3), p=3.3e−03) and ‘Better—depression’ (THC mean: 254.7 (SD: 291.5); β=40.37 (95% CI 15.1 to 65.7), p=1.74e−03) were associated with higher average weekly THC units. Those who started to use for ‘Better—pain’ (THC mean: 250.5 (SD: 337.2); β=20.4 (95% CI: −12.5 to 53.3), p=0.22) and for ‘Better—physical discomfort’ (THC mean: 248.5 (SD: 318.8, median: 136); β=16.2 (95% CI −20.2 to 52.7), p=0.38) were not significantly associated with higher weekly THC unit consumption, although both the high mean levels and high β coefficients indicated a tendency toward higher use (online supplemental tables S5 and S7).

### Associations between RFUC and paranoia, anxiety and depression scores

Linear regressions were conducted to test the associations between the 10 RFUC and paranoia (GPTS^TOTAL^ score), anxiety (GAD-7 score) and depressive symptoms (PHQ-9 score). Including the THC unit measure as a covariate had a marginal impact on the results, with a slight reduction in most effect sizes. Replacing it with frequency of use had an even smaller effect, with no changes in the direction of associations or statistical significance across all regressions (table 2; figure 2; online supplemental table S9).

**Table 2:** Adjusted linear regressions to test the associations between RFUC (Reasons for first using cannabis) and (1) GPTS-tot, (2) GAD-7 and (3) PHQ-9

	GPTS-TOTAL		GAD		PHQ-9	
	β (SE)	P value	β (SE)	P value	β (SE)	P value
Friends Adjusted for THC unit	−0.33 (0.83) 0.25 (0.96)	0.69 0.79	−0.24 (0.2) −0.17 (0.23)	0.23 0.45	−0.02 (0.24) 0.05 (0.28)	0.93 0.85
Family Adjusted for THC unit	3.16 (1.24) 2.15 (1.41)	1.1e−02 **5e−03**	0.76 (0.3) 0.56 (0.3)	1.02e−02 9e−02	1.11 (0.36) 0.82 (0.41)	**1.88e−03** 0.04
Better—physical discomfort Adjusted for THC unit	8.63 (1.59) 8.89 (1.75)	**6.51e−08** **4.12e−07**	0.99 (0.38) 1.07 (0.41)	8.43e−03 **0.01**	1.29 (0.46) 1.42 (0.51)	**5.04e−03** 5.03e−02
Better—pain Adjusted for THC unit	8.14 (1.44) 7.24 (1.59)	**1.63e−08** **5.56e−06**	1.41 (0.34) 1.31 (0.38)	**3.94e−05** **4.75e−04**	1.82 (0.41) 1.57 (0.46)	**1.07e−05** **6.41e−04**
Better—anxiety Adjusted for THC unit	9.56 (1.04) 9.67 (1.16)	**8.1e−20** **1.63e−16**	2.69 (0.25) 2.66 (0.27)	**2.42e−27** **4.35e−22**	3.2 (0.3) 3.07 (0.3)	**1.56e−26** **7e−20**
Better—depression Adjusted for THC unit	9.7 (1.10) 9.12 (1.22)	**1.6e−18** **1.21e−13**	2.38 (0.26) 2.28 (0.29)	**9.11e−20** **3.65e−15**	3.72 (0.31) 3.60 (0.35)	**4.25e−32** **1.38e−24**
Better—psychosis Adjusted for THC unit	20.89 (3.9) 16.5 (4.26)	**9.14e−08** **0.0001**	4.35 (0.93) 4.02 (1)	**2.68e−06** **6.26e−05**	5.26 (1.12) 5.12 (1.22)	**2.74e−06** **3.06e−05**
Curiosity Adjusted for THC unit	−3.49 (0.79) −2.61 (0.93)	**9.32e−06** **0.005**	−0.47 (0.19) −0.44 (0.22)	0.01 4.18e−02	−0.23 (0.23) −0.19 (0.27)	0.3 0.48
Fun Adjusted for THC unit	−3.88 (0.76) −3.71 (0.9)	**3.39e−07** **3.49e−05**	−0.73 (0.18) −0.73 (0.21)	**5.39e−05** **6.04e−04**	−0.55 (0.22) −0.55 (0.26)	1.23e−02 3.29e−02
Boredom Adjusted for THC unit	3.66 (1.17) 3.06 (1.31)	**1.79e−03** 0.019	0.65 (0.28) 0.52 (0.31)	1.97e−02 9.12e−02	1.34 (0.34) 1.09 (0.38)	**6.96e−05** **3.8e−03**

All the analyses were adjusted for sex, age, ethnicity, employment status and years of education. Additional adjustment for THC is also reported. We applied a Bonferroni correction for 10 independent reasons for first using cannabis, setting the significance threshold at p<0.005.

GAD-7, Generalised Anxiety Disorder assessment; GPTS-tot, Green et al Paranoid Thoughts Scale Total Score; PHQ-9, Patient Health Questionnaire for depression; RFUC, reasons for first using cannabis; β, beta coefficient.

## Discussion

To our knowledge, this is the largest cross-sectional study of adult cannabis users to explore how RFUC correlate with reasons for continuing use, and for the first time to investigate if RFUC were associated with standard weekly THC units consumption, paranoia, anxiety and depressed mood, in cannabis users who had not been diagnosed or treated for psychotic disorders. Participants (N=3389 reporting cannabis use; N=2573 current, N=816 past) were young adults (mean age 30.9 years, SD: 9.8), mostly male (62.1%) and predominantly white British/white Other (65.1%). The majority were employed (85.4%) and spent an average of 15.9 years in education. The average age at first cannabis use was 16.7 years, and half (50.9%) used cannabis daily (table 1). The mean weekly standard THC unit consumption was 206 THC units, significantly higher than the 60–80 units reported in the CannTeen study.^22^ This is likely due to the stringent enrolment criteria of the CannTeen study, which excluded, for instance, participants with a diagnosis of cannabis use disorder or any physical health condition deemed problematic by a medical doctor, as well as those using cannabis more than once a week during adolescence (for the adult group). In contrast, our study included a broader range of participants, making our sample more representative of the demographics and clinical characteristics of the London cannabis-using population.

Starting to use cannabis for anxiety or depression was significantly correlated with continuing to use cannabis to be ‘less nervous and anxious’, showing low-to-moderate correlations of r=0.36 and r=0.31, respectively. These correlations are somewhat lower than expected, suggesting that reasons for cannabis use change over time. This aligns with findings from a previous study conducted on patients with first-episode psychosis, which reported a reduction in the strength of endorsed reasons for cannabis use at 3 months and 12 months compared with baseline.^25^ Age at first cannabis use differed across various RFUC. Participants reporting starting use for pain and ‘physical discomfort’ as RFUC initiated use marginally later than those who used it to alleviate psychological distress. Interestingly, the earliest onset was observed among those who reported starting for minor ‘psychotic symptoms (mean: 15.3 (SD: 2.7)) or with ‘family’ (mean: 15.2 (SD: 3.8)).

All RFUC groups had average GAD scores within the mild anxiety range (5–10), except for ‘Better—psychosis’ (mean: 11.1 (SD: 5.3)), which fell within the moderate anxiety category, exceeding the threshold for follow-up or symptom monitoring.^17^ Similarly, most RFUC groups had mean depression in the mild range (5–9), except for ‘Better—pain’ (mean: 10.1 (SD: 7.2)), ‘Better—anxiety’ (mean: 11.1 (SD: 7.2)), ‘Better—depression’ (mean: 11.7 (SD: 7.2))) and ‘Better—psychosis’ (mean: 14.1 (SD: 7)), which fell within the moderate depression severity range (10–14), meeting criteria for referral counselling for follow-up^18^ (online supplemental table S10).

### Comparison with previous research

In a previous case–control study, we found that 75.6% of first-episode psychosis patients and 86.1% of controls reported starting using cannabis because of ‘friends’.^3^ In November 2018, in the UK, medicinal cannabis became legal, and this might be reflected in the slight shift in RFUC, with the percentage of RFUC because of ‘friends’ being slightly lower (69.8%) and a higher proportion of RFUC to feel better compared with the above study. Nevertheless, given that curiosity (62.2%) and fun (52.8%) are, respectively, the second and third most common RFUC, our findings confirm that people are more likely to report using cannabis in a social context and for enhancement.^1^ A novel feature of this study is the ability to distinguish different categories of self-medication motives (RFUC ‘to feel better’). Among these, ‘anxiety’ (15.4%) and ‘depression’ (13.8%) were the most common, followed by ‘pain’ (7.6%) and ‘physical discomfort’ (6%). Although using different methodologies, a recent survey conducted in the USA and Canada on self-reported reasons for medical cannabis use found that pain (53%) was the most common physical health motive, while anxiety (52%) and depression (40%) were the most common mental health ones.^2^ In that study, physical health motives slightly outweighed mental health ones overall. This might be the result of the demographics of the age at first cannabis use in our RFUC sample (16.7 years). At this age, people are less likely to start using cannabis to get relief from physical health problems. However, as reported previously, while the average age at first use does vary among the different RFUC, these variations are not particularly pronounced (online supplemental tables S4 and S6) and, more importantly, in all categories related to using cannabis ‘to feel better’, scores for GPTS^TOTAL^, GAD and PHQ-9 are higher compared with categories like ‘friends’, ‘fun’ and ‘curiosity’. Interestingly, a small US-based survey conducted in 2020 showed that 76% of their sample reported using cannabis to self-medicate from either physical or psychological discomfort.^26^ Our study, conducted in a country where recreational cannabis is still officially illegal, shows that the majority of people report starting to use cannabis in the social context and for enhancement rather than ‘to feel better’. Notably, the RFUC categories ‘Better—pain’ and ‘Better—physical discomfort’ showed weak correlations with all reasons for continued use and were both associated with higher, though not statistically significant, weekly THC unit consumption. A US-based epidemiological survey suggested that people using medical cannabis for pain were more likely to develop patterns of use similar to recreational cannabis users. Additionally, participants with pain were more likely to develop frequent cannabis use compared with people without pain. The authors of the study concluded that adults with pain are a group at higher risk of developing adverse cannabis outcomes.^27^ Indeed, we found that starting for ‘pain’ and ‘physical discomfort’ was both associated with higher levels of psychopathology.

### Limitations and strengths

Participants were recruited through advertisement, potentially leading to a non-entirely representative sample of the London general adult population (≥18 years). Nevertheless, compared with the ethnic breakdown against the latest London census data,^28^ our sample showed an ethnic distribution (online supplemental figures S8 and S9) representative of the ethnic diversity and distribution of the London area. Also, while the census data refers to the overall London population, our sample focused on cannabis users, making it plausible that some of the unrepresented groups were simply less likely to use cannabis, as reported by previous studies, which included data on cannabis use among participants from the London area.^29^

The cross-sectional nature of our study does not allow us to draw any conclusions in terms of causality, particularly regarding the association between RFUC and psychopathology. Nevertheless, it clearly highlights that people who use cannabis to seek relief from either physical or psychological discomfort present with more psychopathology. Furthermore, while sensitivity analyses indicated moderate to high agreement (ranging from κ=0.68 for RFUC ‘fun’ to κ=0.99 for RFUCs for physical discomfort and psychosis) between responses in the online survey and face-to-face assessments for all RFUC variables, recall bias cannot be ruled out, as RFUC data were collected retrospectively through self-report (online supplemental table S2).

Despite these limitations, this study has strengths. Self-administered surveys offered greater anonymity for individuals using illicit substances.^30^ Furthermore, we included 10 distinct RFUC options, which allowed us, compared with previous studies, to capture a broad range of RFUC, including self-medication motives for both psychological and physical discomfort (namely, the ‘to feel better’ categories).

Additionally, a face-to-face assessment was also used for blood for THC measurement and validated our measurement of weekly THC consumption. This is an important strength of our study, which helped us to use a comprehensive and direct standardised measure of THC consumption. Moreover, the standard weekly THC units showed excellent validity when correlated with THC blood concentrations (r=0.77, p=3.08e−13) (online supplemental figure S7). THC units were also associated with RFUC. Those who reported first using cannabis to feel relief from psychotic symptoms had the highest THC consumption (mean: 307.4 (SD: 353.6) THC units), while the group with the lowest THC weekly consumption had started for ‘curiosity’ (mean: 193.6 (SD: 259.7) THC units) (online supplemental table S5).

Another strength of this study is that it was conducted in a general population sample of non-help-seeking cannabis-using individuals living in an area with high levels of cannabis use and an elevated incidence of psychosis.^14 19^ This design helps to identify people for targeted interventions and offers valuable clinical insights.

### Clinical implications

Our findings, for the first time, provide evidence that people who start using cannabis to reduce psychological or physical discomfort differ from those who start for reasons related to socialising, curiosity and having fun. The former report higher average weekly THC unit consumption (and blood levels of THC) and higher levels of paranoia, anxiety and depression. Asking people why they started using cannabis could become an easy and cheap tool, in clinical and non-clinical settings to identify users, including those who are prescribed cannabis, who might benefit from monitoring, support or referral to intervention services to prevent a possible transition from moderately severe psychopathology to potentially disabling anxiety, depression and paranoia.

## Supplementary material

10.1136/bmjment-2025-301810 online supplemental file 1

## Data Availability

Data are available upon reasonable request.
