# Supplementary material for: Are reasons for first using cannabis associated with subsequent cannabis consumption (standard THC units) and psychopathology?
Source: BMJ Ment Health. 2025 Aug 27;28(1):e301810. doi: 10.1136/bmjment-2025-301810 (PMC12382515; doi:10.1136/bmjment-2025-301810)
Supplement: online supplemental file 1 [file bmjment-28-1-s001.docx]

**SUPPLEMENTARY MATERIALS TO ACCOMPANY:**

**Are reasons for first using cannabis associated with subsequent cannabis consumption (standard THC units) and psychopathology?**

Contents

[**SUPPLEMENTARY** 2](#_Toc201222058)

[**1. Methods** 2](#_Toc201222059)

[1.1 Cannabis & Me Study Data Collection 2](#_Toc201222060)

[1.2 Measures: the cannabis use variables and additional cannabis context statements 2](#_Toc201222061)

[1.3 The reasons for first using cannabis (RFUC) variables – validity and recall bias 3](#_Toc201222062)

[**2. Results** 4](#_Toc201222063)

[2.1 Cannabis & Me study recruitment flow chart 4](#_Toc201222064)

[2.2 Participants’ characteristics in the whole sample 5](#_Toc201222065)

[2.3 Reasons for first using cannabis and age at first cannabis use 6](#_Toc201222066)

[2.4 The RFUC variable – analysing the overlapping answers 8](#_Toc201222067)

[2.5 The correlation between RFUC and RCUC 9](#_Toc201222068)

[2.6 The THC unit measure and sensitivity analyses to test the association between the RFUC and the THC unit measure 10](#_Toc201222069)

[2.7 Linear regressions to test the associations between each RFUC and the five outcomes of the study. 13](#_Toc201222070)

[2.8 Multinomial logistic regressions to test the associations between each RFUC and frequency of cannabis use. 14](#_Toc201222071)

[2.8 Associations between each RFUC and 1) GPTS-tot, 2) GAD-7, and 3) PHQ-9. Adding frequency of cannabis use as a covariate. 17](#_Toc201222072)

[2.9 Correlation between THC blood sample analytics with self-reported cannabis use measure. 21](#_Toc201222073)

[2.10 Sample representativeness 22](#_Toc201222074)

[2.11 Sensitivity analyses restricted to those who reported only a single RFUC 25](#_Toc201222075)

[2.12 Full version of Table 2 reporting the full statistics 28](#_Toc201222076)

*The supplementary material has been provided by the authors to give readers additional information about their work*

# **SUPPLEMENTARY**

# **1. Methods**

## 1.1 Cannabis & Me Study Data Collection

Participants were recruited through an online survey promoted via targeted advertisements on social media and cannabis-related forums, specifically aimed at adults (≥18 years old). The recruitment strategy sought to ensure diversity and representativeness among cannabis users and non-users. Individuals with a prior diagnosis of, or treatment history for a psychotic disorder were excluded.

Upon accessing the survey, participants were first presented with a study information sheet and consent form. Only those who provided informed consent could proceed. To maintain data quality, only fully completed questionnaires with mandatory responses were accepted, except for ‘employment status’ and the standard THC unit measure. Employment status was dichotomised into ‘employed’ and ‘unemployed,’ requiring the removal of ‘None of the above’ and ‘Prefer not to say’ responses. The THC unit measure, which had 25.3% missing data, was also derived from the non-mandatory question, ‘What type of cannabis do you use?’.

## 1.2 Measures: the cannabis use variables and additional cannabis context statements

As in previous studies, our cannabis use measures were collected using the Cannabis Experience Questionnaire modified version (CEQ_EU-GEImv_)^1^.

None of the materials used for the participants' recruitment referred to cannabis or its potential role as a risk factor for psychotic disorder. This applied to all stages of the Cannabis & Me study, both the online survey and the Study 1&2 face-to-face assessment. Participants were asked if they had ever used cannabis. If yes, they were asked to answer questions about their pattern of use, including the type of cannabis, allowing them to report the “street” name of the cannabis they used with no reference to its potency.

As suggested in the recent commentary written by Cousjin and colleagues^2^, we have added a table which aims to provide key information on the cannabis context in which the study was conducted. We welcome their suggestion to help characterise heterogeneity in context across studies, potentially aiming at improving data synthesis.

**Table S1.** *Statements on cannabis context*

| **Cannabis Research Context Statements** | |
| --- | --- |
| Location | This study was conducted in the London area. |
| Year | The analyses are based on data collected between 30/03/2022 and 31/07/2024 |
| Cannabis Policy | Recreational cannabis is still illegal in the UK, under the law, and it is classified as a Class B drug, making its unauthorised possession, distribution, cultivation, or sale illegal. Since 2018, Cannabis-based products for Medicinal Use are allowed to be prescribed by specialist doctors when deemed clinically appropriate. |
| Common method of administration | The majority of users smoked “joints” with added tobacco. |
| Average regional cannabinoid content | According to Potter et al 2018, in the London area, the average THC concentration in 2016 was 15.5%^3^. In our sample, we found an average standard THC unit of 206 (SD = 268). |
| Regional prevalence of Cannabis use and frequency | According to the Crime Survey for England and Wales (CSEW), the proportion of 16 to 59-year-olds reporting lifetime cannabis use was 31.2% in the period between October 2021 and June 2022. Of these, 38.8% reported daily cannabis use^4^. In our subsample of people with lifetime cannabis use, the prevalence of daily cannabis use was 50.9%. |

## 1.3 The reasons for first using cannabis (RFUC) variables – validity and recall bias

In our previous paper on reasons for first using cannabis, we tested the reliability of three reasons for first using cannabis by comparing the baseline and follow-up data of both the Genetic and Psychosis Study (GAP) and from the London sample of the EU-GEI study to rule out possible recall bias issues. We found a good level of agreement for all our variables, both in cases and controls^5^.

In our current study, we have used a modified version of the Cannabis Experience Questionnaire CEQ_mv_ and asked participants to provide up to 10 reasons for first using cannabis. This aimed to consider the highest possible number of reasons to start and distinguish different features of 1) self-medication/coping and 2) enhancement/social motivations.

Some of our online survey respondents later participated in a subsequent part of the Cannabis and Me Study, namely, Study 1&2 face-to-face assessment. Because they were again asked to complete the Cannabis Experience Questionnaire, we had the opportunity to compare the RFUC answers at different moments. More precisely, we collected data on 87 participants with either high or low levels of paranoia, who, after completing the online survey, were contacted to complete a face-to-face assessment.

Recall bias cannot be entirely ruled out in this case, given the relatively short time gap between the two assessments (less than one year). However, the results indicate a moderate to high level of agreement for all the variables, with Cohen’s Kappa coefficients ranging from 67.8% to 98.9%. This suggests that participants’ recall of their initial responses was largely accurate over the period between assessments.

Overall, the substantial agreement across most categories supports the reliability of our self-reported data and indicates minimal recall bias, affirming the robustness of our measures.

**Table S2. *Testing the reliability of our different RFUC variables***

|  | Kappa coefficient |
| --- | --- |
| RFUC “friends” | κ = 0.76 |
| RFUC “family” | κ = 0.93 |
| RFUC “better (physical discomfort)” | κ = 0.99 |
| RFUC “better (pain)” | κ = 0.97 |
| RFUC “better (anxiety)” | κ = 0.84 |
| RFUC “better (depression)” | κ = 0.87 |
| RFUC “better (psychosis)” | κ = 0.99 |
| RFUC “curiosity” | κ = 0.71 |
| RFUC “fun” | κ = 0.68 |
| RFUC “boredom” | κ = 0.78 |

|  |
| --- |
|  |

# **2. Results**

## 2.1 Cannabis & Me study recruitment flow chart

As outlined in the main text, data collection for the CAMe online survey was conducted using the <https://www.onlinesurveys.ac.uk/> platform. On 31/07/2024, version 2 of the online survey ceased operating, switching to version 3, which is currently being used to further expand the sample of never-users. Thus, this study uses incomplete data but retains sufficient statistical power for analyses among people with lifetime cannabis use (both past and current). Below, we reported the recruitment study flow chart for the study.

**Figure S1.** *Recrutiment flow chart*


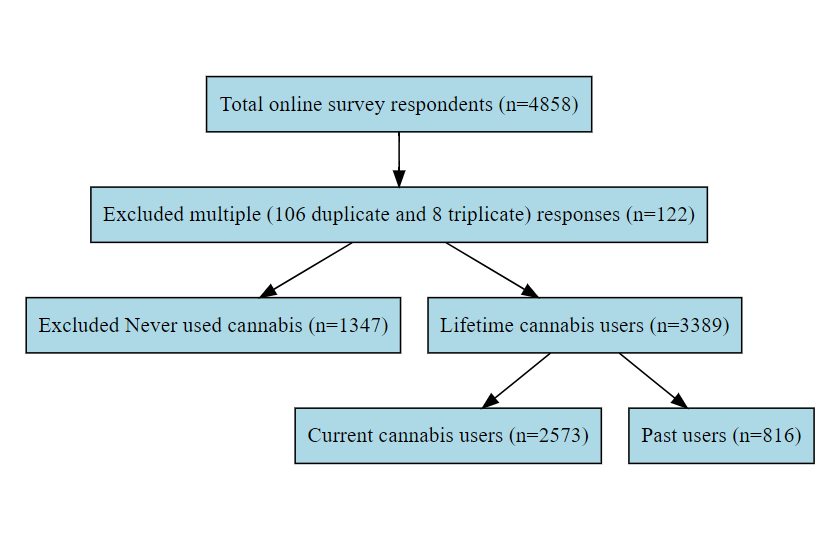


## 2.2 Participants’ characteristics in the whole sample

**Table S3. *Participants’ characteristics in the whole sample (n=4,736 cannabis users and non-users)***

|  | **Variable** | **Statistics** | **Descriptor** |
| --- | --- | --- | --- |
| **Demographics** | Age | M (SD), Median (IQR) | 31.8 (10.5), 29 (13) |
|  | Sex | Males (%) | 2,632 (55.6) |
|  | Ethnicity | White/White other (%) | 2,891 (61) |
|  | Employment status | Employed (%) | 3,977 (86.9) |
|  | Years of education | M (SD), Median (IQR) | 16.3 (3.9), 17 (5) |
| **Cannabis use** | Lifetime Cannabis use | Yes (%) | 3,389 (71.6%) |
|  | Frequency of use | Daily use (%) | 1,719 (36.3%) |
|  | Age first tried | M (SD), Median (IQR) | 16.7 (5.6), 16 (4) |
| **Psychopathology** | GAD-7 | M (SD), Median (IQR) | 5.8 (5.3), 5 (7) |
|  | PHQ-9 | M (SD), Median (IQR) | 7.2 (6.4), 6 (9) |
|  | GPTS-A | M (SD), Median (IQR) | 26.9 (11.7), 23 (14) |
|  | GPTS-B | M (SD), Median (IQR) | 22.7 (11.4), 17 (8) |
|  | GPTS-TOT | M (SD), Median (IQR) | 49.6 (21.8), 41 (21) |

M = mean; SD = Standard deviation; IQR = Interquartile Range; GAD-7 = Generalised Anxiety Disorder assessment; PHQ-9 = Patient Health Questionnaire for depression; GPTS = Green et al Paranoid Thoughts Scale; GPTS-A = ideas of reference; GPTS-B = persecution; GPTS-TOT = GPTS total score.

**Figure S2. *Prevalence of lifetime cannabis use in our sample of online survey respondents***


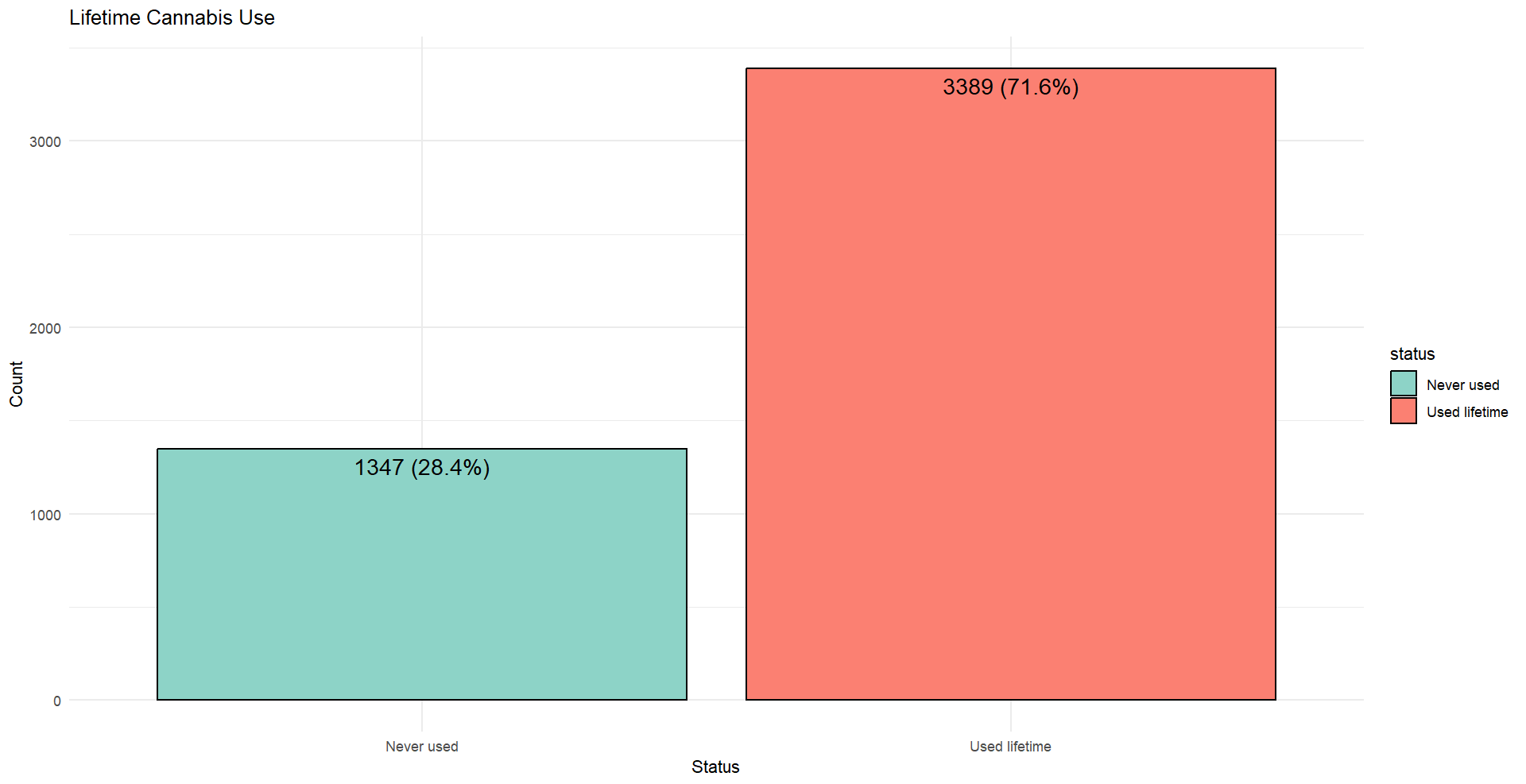


## 2.3 Reasons for first using cannabis and age at first cannabis use

In line with our previous study^5^, we found that people are generally more likely to report starting to use cannabis in the social context and for hedonistic reasons rather than to self-medicate or to cope with negative experiences. It could be argued that this is mostly the result of the demographics of our sample and it does not reflect the broader motivations to use cannabis in the whole population of cannabis users. As previously reported, the average age at first use in our sample is 16.7 years, aligning closely with the average onset age reported in other UK studies. At this age, individuals are more likely to begin using cannabis in social settings rather than for reasons like pain relief or alleviating physical discomfort. However, it's important to note that, firstly, while the age at first use does vary among the different reasons for initiation, these variations are not particularly pronounced. Secondly, and more importantly, in all categories related to using cannabis 'to feel better’, scores for GPTS-tot, GAD, and PHQ-9 are higher compared to categories like friends, fun, and curiosity. This suggests that although the age at first use provides some context, it does not fully explain the influence that the reasons for starting have on later patterns of cannabis use and subclinical psychopathology. These findings have significant epidemiological and clinical implications, indicating that the motivations behind initial cannabis use can impact subsequent mental health.

**Table S4. *Descriptive statistics assessing the association between RFUC and age at first use***

|  |  |  |
| --- | --- | --- |
| **RFUC** | Statistics, age at first use |  |
|  | M (SD), Median (IQR) | Statistics and p-value |
| **Friends** | 16.43 (4.46), 16 (4) | U = 1.86; p = 0.06 |
| **Family** | 15.23 (3.83), 15 (3) | **U = 9.75; p < 0.001** |
| **Better—physical discomfort** | 17.98 (9.18), 16 (4) | U = -0.6; p = 0.54 |
| **Better—pain** | 17.83 (8.89), 16 (4) | U = -0.02; p = 0.98 |
| **Better—anxiety** | 16.78 (7.68), 16 (4) | U = 1.48; p = 0.14 |
| **Better—depression** | 16.51 (4.62), 16 (4) | **U = 2.61; p = 0.009** |
| **Better—Psychosis** | 15.31 (2.72), 15 (3.5) | U = 1.52; p = 0.12 |
| **Curiosity** | 16.62 (5.78), 16 (4) | U = -0.14; p = 0.89 |
| **Fun** | 16.35 (4.85), 16 (4) | **U = 3.98; p < 0.001** |
| **Boredom** | 16.05 (8.07), 15 (3) | **U = 6; p < 0.001** |

*U = Mann-Whitney U test*

## 2.4 The RFUC variable – analysing the overlapping answers

As can be seen in the figure below (see eFigure 3), participants were able to provide more than one answer to the question ‘Why did you first try cannabis?’. Figure S3 limits the display to the top 20 combinations to improve readability.

**Figure S3. *Prevalence of the top 20 reasons for first using cannabis and overlaps in the working sample of people with lifetime cannabis use***


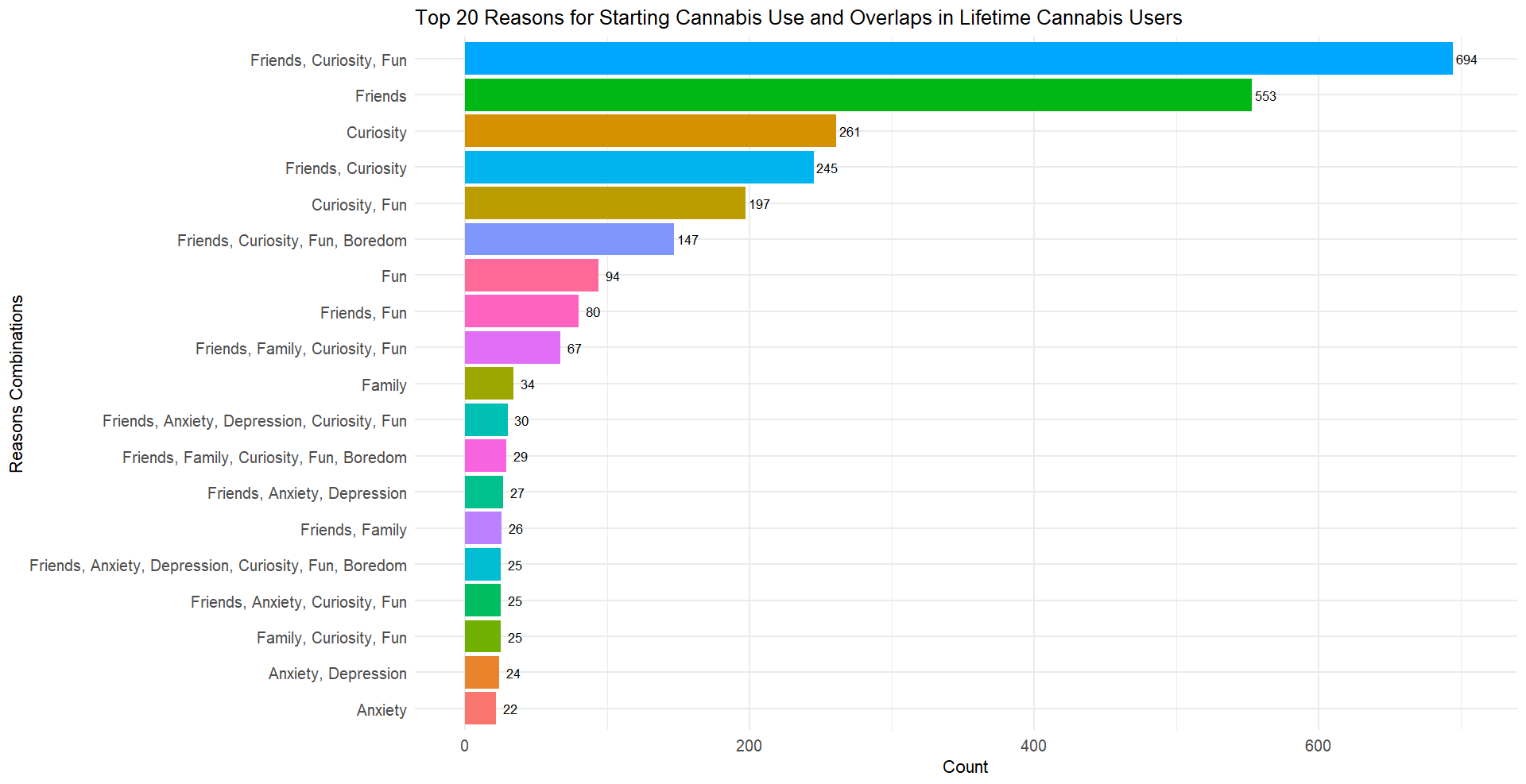


## 2.5 The correlation between RFUC and RCUC

**Figure S4. *Correlation grid showing the correlation between reasons for first using cannabis and reasons for continuing to use cannabis***


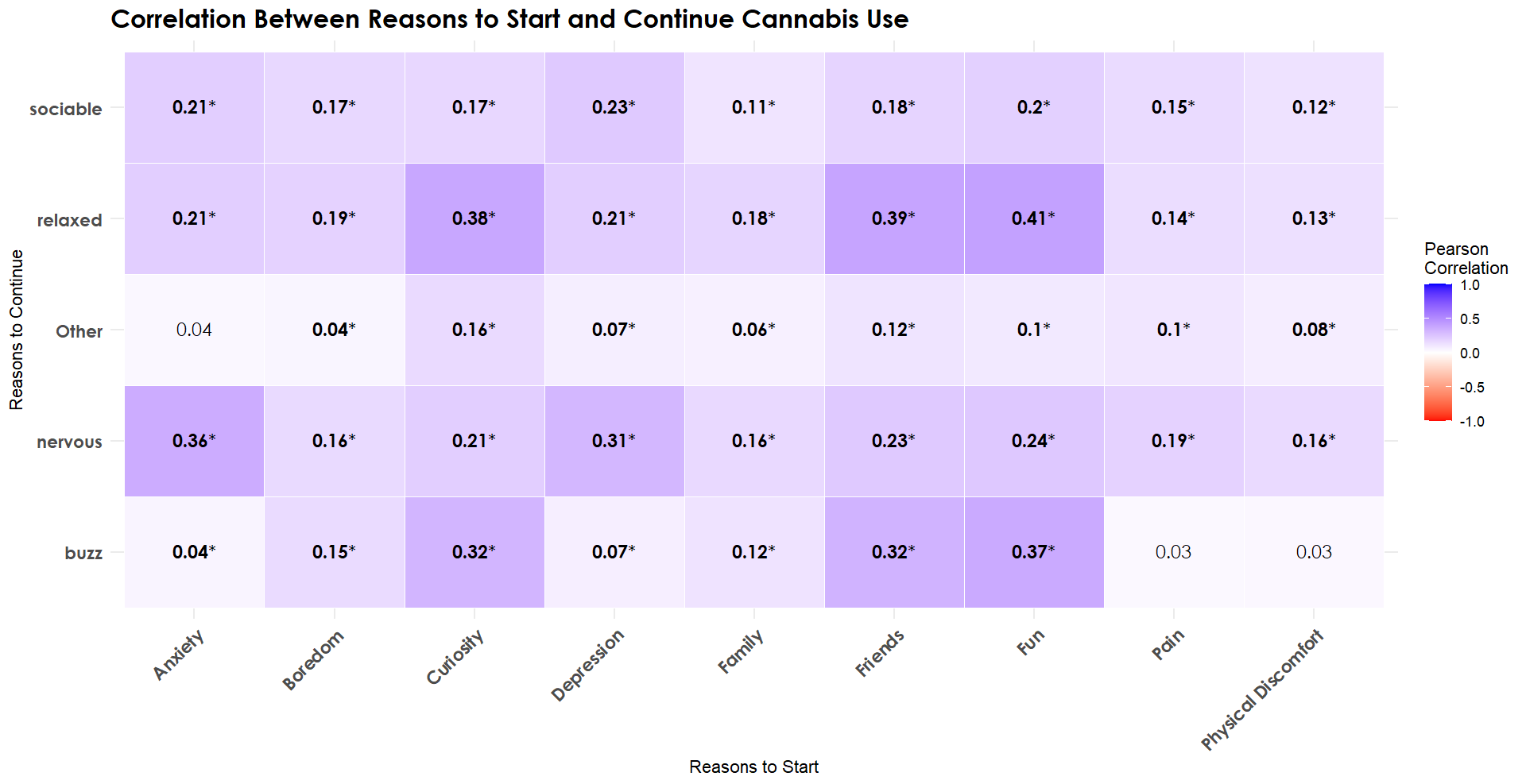


We applied a Bonferroni correction for 10 independent reasons for first using cannabis, setting the significance threshold at p<0.005. P-values below this threshold are marked with an asterisk.

## 2.6 The THC unit measure and sensitivity analyses to test the association between the RFUC and the THC unit measure

As already discussed in the methods section of the manuscript, the standard THC unit measure was generated as a result of the frequency of cannabis variable not being able to capture other aspects of harmful consumption of cannabis, such as the potency of use. Therefore, we also collected self-reported information on the type of cannabis used and the quantity in grams used per week. We combined these two measures to estimate dose-related THC consumption using standard THC units (one unit = 5 mg THC), consistent with the National Institutes of Health reporting recommendations to report using standard THC units^6^.

Using this method, we managed to calculate a measure of THC in 2,532 people with lifetime cannabis cannabis use. This left us with 857 missing observations (25.3%). More precisely, participants reporting past cannabis use were characterised by the highest missing rate (66.6%), whereas people with current cannabis use had 12.01% of missing observations on the standard THC unit.

Next, we calculated the distribution of the THC unit across our two subgroups of cannabis-using participants.

For participants with current cannabis use, the mean THC unit was slightly higher at 224 (SD = 283, Median = 132, IQR = 230).

For participants with past cannabis use, the mean THC unit was considerably lower at 82.5 (SD = 140, Median = 30, IQR = 94.2).

Given that the standard THC unit had high variability, as indicated by large SD, we applied winsorization. This is a method used for handling extreme values in continuous data by capping values at a certain threshold.

Given the high level of missing data in the standard THC unit variable (25.3%), we evaluated missing data patterns by checking if missing values are systematically associated with key demographics. Since the large majority (3,502 participants) had complete data across the specified variables, this suggested that there was not a widespread or complex pattern of missingness for these variables. This confirmed patterns of non-random missingness.

Therefore, under the assumption that the data were Missing at Random (MAR), we applied Multiple Imputation by Chained Equations (MICE). MICE was implemented using: 1) Predictive mean matching (pmm) for continuous variables; 2) logistic regression for binary categorical variables; 3) multinomial logistic regression for categorical variables with more than two levels. Five imputations were performed (m = 5), with random seed 123 to ensure reproducibility. Diagnostic checks confirmed the convergence of the algorithm and consistency between observed and imputed data distributions.

More precisely, we compared the distribution of imputed values to observed values for the THC unit variable, confirming that imputed values aligned with observed patterns. See Figures S5 and S6.

**Figure S5. *Plot showing the distribution of imputed THC unit values across the five imputations.***


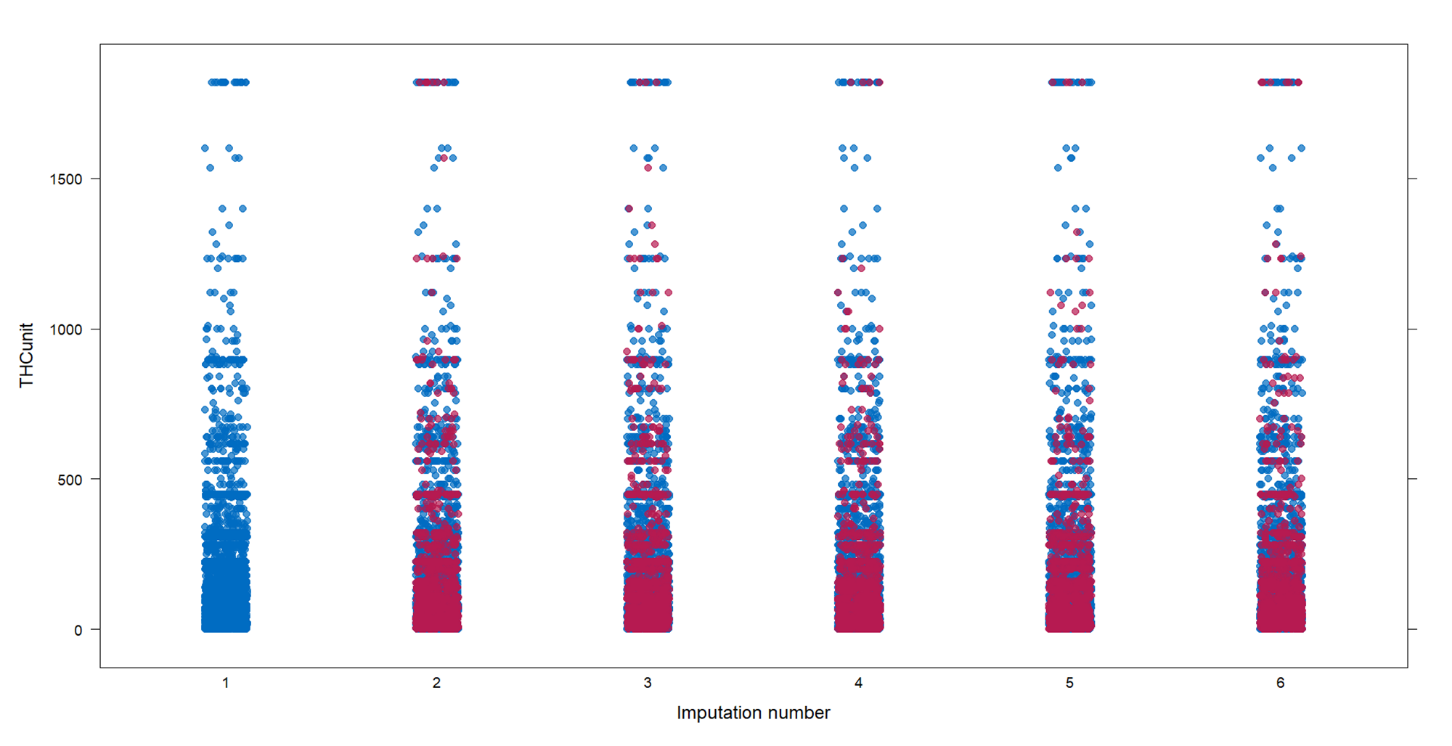


*In the plot above, blue dots represent the original points, and red dots represent the imputed values (5 imputations depicted in columns 2 to 6).*

This plot shows consistency across impotations and overlap between observed and imputed data.

**Figure S6. Convergence plot examining the iteration history for convergence diagnostics.**


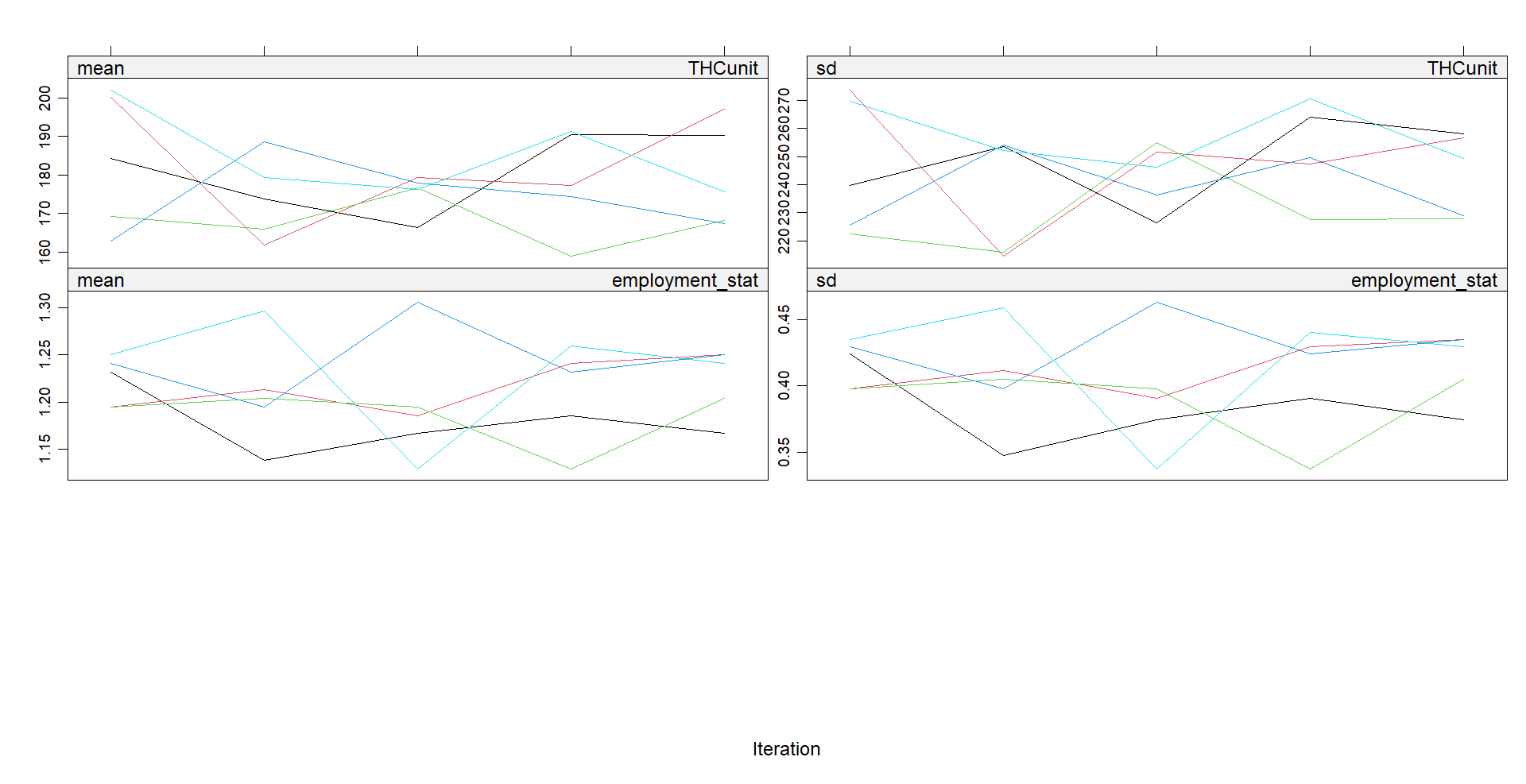


The plot suggests the stability of parameters and consistency across imputation.

Additionally, we conducted simple descriptive analyses to test the associations between the RFUC and the THC unit measure as measured in weekly standard THC units.

**Table S5. Descriptive associations between each RFUC and the THC unit measure**

| **RFUC** | Statistics, THC unit |  |  |
| --- | --- | --- | --- |
|  | M (SD), Median (IQR) | | Statistics and p-value |
| **Friends** | 196.2 (261.7), 112 (200) | U = 634593 ; **p = 4.97e-04** | |
| **Family** | 286.9 (357), 154 (295) | U = 387507**; p 4.25e-07** | |
| **Better—physical discomfort** | 248.5 (318.8), 136 (236.95) | U = 233099; p = 5.86e-03 | |
| **Better—pain** | 250.5 (337.2), 140 (236.95) | U = 282490; **p = 6.54e-03** | |
| **Better—anxiety** | 248 (294.1), 153.65 (250) | U = 536901.5; **p = 1.55e-07** | |
| **Better—depression** | 254.7 (291.5), 150 (250) | U = 508042.5; **p = 1.78e-09** | |
| **Better—Psychosis** | 307.4 (353.6), 192 (336) | U = 43976.5; p = 7.16e-03 | |
| **Curiosity** | 193.6 (259.7), 112 (184) | U = 688458.5; **p = 2.92e-04** | |
| **Fun** | 194.8 (255.4), 112 (189.3) | U = 756535.5; p = 3.14e-02 | |
| **Boredom** | 240.2 (277.9), 154 (243) | U = 419270; **p = 7.64e-05** | |

*U = Mann-Whitney U test*. We applied a Bonferroni correction for 10 independent reasons for first using cannabis, setting the significance threshold at p<0.005. P-values below this threshold are marked with an asterisk.

## 2.7 Linear regressions to test the associations between each RFUC and the five outcomes of the study.

The Tables below display the outcome of the linear regression models, which are present in the main manuscript as Figure 2.

**Table S6. *Linear regressions testing the association between RFUC and age at first cannabis use***

|  |  | **Age at first cannabis use*** | | |
| --- | --- | --- | --- | --- |
|  | β (SE) | | 95% CI | p-value |
| **Friends** | -1.1 (0.21) | | -1.52, -0.68 | **3.17e-07** |
| **Family** | -1.58 (0.32) | | -2.21, -0.95 | **9.5e-07** |
| **Better—physical discomfort** | 1.72 (0.41) | | 0.91, 2.52 | **2.87e-05** |
| **Better—pain** | 1.46 (0.37) | | 0.73, 2.19 | **9.42e-05** |
| **Better—anxiety** | 0.53 (0.27) | | -0.002, 1.06 | 0.051 |
| **Better—depression** | 0.09 (0.29) | | -0.47, 0.65 | 0.75 |
| **Better—psychosis** | -0.84 (1.02) | | -2.86, 1.16 | 0.41 |
| **Curiosity** | -0.27 (0.2) | | -0.67, 0.13 | 0.19 |
| **Fun** | -0.67 (0.2) | | -1.05, -0.28 | **6.57e-04** |
| **Boredom** | -0.41 (0.3) | | -1, 0.19 | 0.18 |

β = beta coefficient; SE = Standard Error. *All the analyses were adjusted for sex, age, ethnicity, employment status, and years of education. We applied a Bonferroni correction for 10 independent reasons for first using cannabis, setting the significance threshold at p<0.005. P-values below this threshold are marked with an asterisk.

**Table S7. Adjusted linear regressions to test the associations between RFUC and the winsorised THC unit**

|  |  | **THC Unit** | | | **THC Unit – IMPUTED MODEL** | | |
| --- | --- | --- | --- | --- | --- | --- | --- |
|  | β (SE) | | 95% CI | p-value | β (SE) | 95% CI | p-value |
| **Friends** | -18.5 (11.3) | | -40.6, 3.6 | 0.1 | -23.9 (9.6) | -42.7, -5.1 | 1.3e-02 |
| **Family** | 82.6 (16.5) | | 50.3, 114.9 | **5.95e-07** | 87.43 (14.3) | 59.4, 115.5 | **1.22e-09** |
| **Better—physical discomfort** | 29 (20.8) | | -11.8, 69.8 | 0.16 | 16.24 (18.6) | -20.2, 52.7 | 0.38 |
| **Better—pain** | 22.2 (18.8) | | -14.6, 59 | 0.24 | 20.42 (16.8) | -12.5, 53.3 | 0.22 |
| **Better—anxiety** | 44.1 (13.9) | | 16.9, 71.3 | **1.52e-03** | 36.22 (12.3) | 12.1, 60.3 | **3.3e-03** |
| **Better—depression** | 53.3 (14.5) | | 24.9, 81.7 | **2.44e-04** | 40.37 (12.9) | 15.1, 65.7 | **1.74e-03** |
| **Better—psychosis** | 59.7 (50.4) | | -39.1, 158.5 | 0.24 | 53.14 (46.4) | -37.8, 144.1 | 0.25 |
| **Curiosity** | -17.3 (11) | | -38.9, 4.3 | 0.11 | -15.04 (9.2) | -33.1, 3 | 0.1 |
| **Fun** | -12.7 (10.6) | | -33.5, 8.1 | 0.23 | -21.7 (8.9) | -39.1, -4.3 | 1.43e-02 |
| **Boredom** | 40.8 (15.5) | | 10.4, 71.2 | **8.32e-03** | 34.1 (13.7) | 7.2, 61 | 1.3e-02 |

β = beta coefficient; SE = Standard Error. *All the analyses were adjusted for sex, age, ethnicity, employment status, and years of education. In all our analyses, we applied a winsorised version of the THC unit. We applied a Bonferroni correction for 10 independent reasons for first using cannabis, setting the significance threshold at p<0.005. P-values below this threshold are marked with an asterisk.

## 2.8 Multinomial logistic regressions to test the associations between each RFUC and frequency of cannabis use.

**Table S8. *Multinomial logistic regressions testing the association between RFUC and frequency of cannabis use***

| Term | Frequency of Cannabis Use | | RRR (95% CI) | | P-value | Term | Frequency of Cannabis Use | | RRR (95% CI) | | | P-value |
| --- | --- | --- | --- | --- | --- | --- | --- | --- | --- | --- | --- | --- |
| **Friends** | Once or twice | (reference) | |  | |  | | Once or twice | | (reference) |  | |
|  | Monthly or less | 0.94 (0.63—1.4) | | 0.75 | |  | | Monthly or less | | 3.91 (1.33—11.52) | 0.013 | |
|  | Weekly or less | 0.72 (0.51—1.02) | | 0.07 | | **Better—pain** | | Weekly or less | | 7.93 (3.14—19.98) | **<0.001** | |
|  | More than once a week | 0.53 (0.4—0.71) | | **<0.001** | |  | | More than once a week | | 8.02 (3.32—19.37) | **<0.001** | |
|  | Daily | 0.54 (0.42—0.69) | | **<0.001** | |  | | Daily | | 9.07 (3.9—21.11) | **<0.001** | |
| **Family** | Once or twice | (reference) | |  | |  | | Once or twice | | (reference) |  | |
|  | Monthly or less | 1.81 (0.93—3.51) | | 0.08 | |  | | Monthly or less | | 1.96 (1.07—3.57) | 0.029 | |
|  | Weekly or less | 1.38 (0.73—2.59) | | 0.32 | | **Better—anxiety** | | Weekly or less | | 2.29 (1.35—3.88) | **0.002** | |
|  | More than once a week | 2.03 (1.21—3.43) | | 0.008 | |  | | More than once a week | | 2.38 (1.48—3.83) | **<0.001** | |
|  | Daily | 3.31 (2.09—5.42) | | **<0.001** | |  | | Daily | | 4.29 (2.81—6.54) | **<0.001** | |
| **Better—physical discomfort** | Once or twice | (reference) | |  | |  | | Once or twice | | (reference) |  | |
|  | Monthly or less | 4.58 (1.32—15.9) | | 0.017 | |  | | Monthly or less | | 2.07 (0.92—4.65) | 0.077 | |
|  | Weekly or less | 10.52 (3.58—30.94) | | **<0.001** | |  | | Weekly or less | | 2.84 (1.42—5.68) | **0.003** | |
|  | More than once a week | 7.94 (2.77—22.78) | | **<0.001** | | **Better—depression** | | More than once a week | | 5.24 (2.85—9.63) | **<0.001** | |
|  | Daily | 8.99 (3.25—24.78) | | **<0.001** | |  | | Daily | | 7.44 (4.18—13.24) | **<0.001** | |
| **Better—psychosis** | Once or twice | (reference) | |  | |  | | Once or twice | | (reference) |  | |
|  | Monthly or less | NA (NA—NA) | | 1 | |  | | Monthly or less | | 1.76 (0.92—3.35) | 0.087 | |
|  | Weekly or less | NA (NA—NA) | | NA | | **Boredom** | | Weekly or less | | 1.97 (1.12—3.47) | 0.02 | |
|  | More than once a week | NA (NA—NA) | | NA | |  | | More than once a week | | 2.22 (1.34—3.66) | **0.002** | |
|  | Daily | NA (NA—NA) | | NA | |  | | Daily | | 3 (1.9—4.74) | **<0.001** | |
| **Curiosity** | Once or twice | (reference) | |  | |  | |  | |  |  | |
|  | Monthly or less | 0.78 (0.55—1.09) | | 0.15 | |  | |  | |  |  | |
|  | Weekly or less | 0.83 (0.61—1.13) | | 0.23 | |  | |  | |  |  | |
|  | More than once a week | 0.83 (0.64—1.09) | | 0.18 | |  | |  | |  |  | |
|  | Daily | 0.77 (0.61—0.96) | | 0.022 | |  | |  | |  |  | |
| **Fun** | Once or twice | (reference) | |  | |  | |  | |  |  | |
|  | Monthly or less | 1.29 (0.93—1.8) | | 0.13 | |  | |  | |  |  | |
|  | Weekly or less | 0.97 (0.72—1.3) | | 0.84 | |  | |  | |  |  | |
|  | More than once a week | 1.37 (1.07—1.77) | | 0.013 | |  | |  | |  |  | |
|  | Daily | 1.15 (0.93—1.43) | | 0.2 | |  | |  | |  |  | |

*These analyses serve as sensitivity analyses for our THC unit measure. All the analyses were adjusted for age, sex, ethnicity, employment status, and years of education. RRR = Relative Risk Ratio.* We applied a Bonferroni correction for 10 independent reasons for first using cannabis, setting the significance threshold at p<0.005. P-values below this threshold are marked with an asterisk. *Multinomial logistic regressions were conducted using STATA-17. NA = Not Applicable. Results for ‘Better—psychosis’ NA due to fewer observations.*

## 2.8 Associations between each RFUC and 1) GPTS-tot, 2) GAD-7, and 3) PHQ-9. Adding frequency of cannabis use as a covariate.

To ensure additional robustness, similarly to what we did in **Table S8**, we conducted a sensitivity analysis replacing the stand THC unit measure with the frequency of cannabis. Therefore, below we conducted the same analyses as those displayed in Table 4 only changing the covariate. As we did previously, all regressions were adjusted for age, sex, ethnicity, years of education, and employment status. For each RFUC, we added a composite measure of the frequency of cannabis use to see if that changed the observed associations. Again, we only observed a small reduction in the effect size.

**Table S9. Adjusted linear regressions to test the associations between RFUC and 1) GPTS-tot, 2) GAD-7, and 3) PHQ-9**

|  | **GPTS-TOTAL** | | **GAD** | | **PHQ-9** | |
| --- | --- | --- | --- | --- | --- | --- |
|  | β (SE) | p-value | β (SE) | p-value | β (SE) | p-value |
| **Friends**  **Adjusted for frequency of use** | -0.33 (0.83)  -0.02 (0.83) | 0.69  0.98 | -0.24 (0.2)  -0.22 (0.2) | 0.23  0.27 | -0.02 (0.24)  0.07 (0.24) | 0.93  0.76 |
| **Family**  **Adjusted for frequency of use** | 3.16 (1.24)  3.03 (1.25) | 1.1e-02  1.5e-02 | 0.76 (0.3)  0.68 (0.3) | 1.02e-02  2.25e-02 | 1.11 (0.36)  0.94 (0.36) | **1.88e-03**  8.59e-03 |
| **Better—physical discomfort**  **Adjusted for frequency of use** | 8.63 (1.59)  8.68 (1.59) | **6.51e-08**  **5.21e-08** | 0.99 (0.38)  1.04 (0.38) | 8.43e-03  6.33e-03 | 1.29 (0.46)  1.23 (0.46) | 5.04e-03  7.39e-03 |
| **Better—pain**  **Adjusted for frequency of use** | 8.14 (1.44)  8.19 (1.44) | **1.63e-08**  **1.33e-08** | 1.41 (0.34)  1.44 (0.34) | **3.94e-05**  **2.5e-05** | 1.82 (0.41)  1.76 (0.41) | **1.07e-05**  **2.14e-05** |
| **Better—anxiety**  **Adjusted for frequency of use** | 9.56 (1.04)  9.54 (1.05) | **8.1e-20**  **1.78e-19** | 2.69 (0.25)  2.63 (0.25) | **2.42e-27**  **1.47e-26** | 3.2 (0.3)  3.06 (0.3) | **1.56e-26**  **4.2e-24** |
| **Better—depression**  **Adjusted for frequency of use** | 9.7 (1.10)  9.72 (1.11) | **1.6e-18**  **2.83e-18** | 2.38 (0.26)  2.42 (0.26) | **9.11e-20**  **5.75e-20** | 3.72 (0.31)  3.67 (0.31) | **4.25e-32**  **8.79e-31** |
| **Better—psychosis**  **Adjusted for frequency of use** | 20.89 (3.9)  20.77 (3.88) | **9.14e-08**  **9.38e-08** | 4.35 (0.93)  4.3 (0.92) | **2.68e-06**  **3.33e-06** | 5.26 (1.12)  5.07 (1.11) | **2.74e-06**  **5.32e-06** |
| **Curiosity**  **Adjusted for frequency of use** | -3.49 (0.79)  -3.24 (0.78) | **9.32e-06**  **3.72e-05** | -0.47 (0.19)  -0.44 (0.19) | 0.01  1.79e-02 | -0.23 (0.23)  -0.71 (0.22) | 0.3  0.46 |
| **Fun**  **Adjusted for frequency of use** | -3.88 (0.76)  -3.73 (0.76) | **3.39e-07**  **9.05e-07** | -0.73 (0.18)  -0.7 (0.18) | **5.39e-05**  **1.05e-04** | -0.55 (0.22)  -0.51 (0.22) | 1.23e-02  1.86e-02 |
| **Boredom**  **Adjusted for frequency of use** | 3.66 (1.17)  3.46 (1.17) | **1.79e-03**  **3.29e-03** | 0.65 (0.28)  0.58 (0.28) | 1.97e-02  3.76e-02 | 1.34 (0.34)  1.2 (0.34) | **6.96e-05**  **3.75e-04** |

β = beta coefficient; SE = Standard Error. **p* ≤ 0.01 ****p* ≤ 0.001. All the analyses were adjusted for sex, age, ethnicity, employment status, and years of education. Additional adjustment for frequency of cannabis use is also reported. We applied a Bonferroni correction for 10 independent reasons for first using cannabis, setting the significance threshold at p<0.005. P-values below this threshold are marked with an asterisk.

Additionally, we conducted simple descriptive analyses to test the associations between the RFUC and 1) GPTS-TOTAL, 2) GAD, and 3) PHQ-9 measures.

**Table S10. Descriptive associations between each RFUC and 1) GPTS-tot, 2) GAD-7, and 3) PHQ-9.**

| **RFUC** | Statistics, GPTS-TOTAL | | Statistics, GAD |  | | Statistics, PHQ-9 | |  |  |
| --- | --- | --- | --- | --- | --- | --- | --- | --- | --- |
|  | M (SD), Median (IQR) | Statistics and p-value | M (SD), Median (IQR) | | Statistics and p-value | M (SD), Median (IQR) | Statistics and p-value | |  |
| **Friends** | 50.4 (22.2), 41 (24) | U = 1174271; p = 1.69e-01 | 6 (5.2), 5 (7) | | U = 1181463; p = 2.7e-01 | 7.7 (6.4), 6 (9) | U = 1192849; p = 5.06e-01 | |  |
| **Family** | 54.8 (25.9), 44 (29) | U = 596646; **p 8.51e-04** | 6.8 (5.5), 6 (9) | | U = 586919; **p = 5.39e-03** | 9 (6.6), 8 (10) | U = 607116; **p = 8.19e-05** | |  |
| **Better—physical discomfort** | 60.3 (28.3), 51 (39.3) | U = 396163; **p =** **1.37e-07** | 7.4 (5.6), 6 (8) | | U = 375366; **p = 1.85e-04** | 9.7 (6.7), 9 (11) | U = 384720; **p = 9.61e-06** | |  |
| **Better—pain** | 59.8 (28.2), 50 (41) | U = 486943; **p = 2.03e-08** | 7.8 (5.9), 7 (9) | | U = 474975; **p = 1.41e-06** | 10.1 (7.2), 9 (11) | U = 487230; **p = 1.78e-08** | |  |
| **Better—anxiety** | 60.3 (27.5), 52 (37) | U = 958012; **p = 8.82e-25** | 8.8 (5.7), 8 (9) | | U = 1000687; **p = 3.27e-35** | 11.1 (7.2), 10 (11) | U = 991905; **p = 7.63e-33** | |  |
| **Better—depression** | 61.2 (27.8), 53 (36) | U = 886482; **p = 9.6e-25** | 8.6 (5.8), 7 (9) | | U = 895185; **p = 7.06e-27** | 11.7 (7.2), 11 (11) | U = 939719; **p = 1.45e-38** | |  |
| **Better—Psychosis** | 75.2 (36.6), 67 (48) | U = 75843; **p = 1.12e-05** | 11.1 (5.3), 12 (8) | | U = 78518; **p = 9.75e-07** | 14.1 (7), 14 (9) | U = 78530; **p = 9.98e-07** | |  |
| **Curiosity** | 49.3 (20.9), 41 (20.5) | U = 1273602; **p = 6.29e-03** | 5.9 (5.1), 5 (7) | | U = 1298773; p = 6.34e-02 | 7.7 (6.3), 6 (8) | U = 1326517; p = 4.16e-01 | |  |
| **Fun** | 49.1 (20.5), 41 (21) | U = 1353674; **p = 6.21e-03** | 5.76 (5.1), 5 (6) | | U = 1348224; **p = 3.35e-08** | 7.6 (6.3), 6 (8) | U = 1390662; p = 1.51e-01 | |  |
| **Boredom** | 54.9 (24), 47 (31) | U = 677098; **p = 1.1e-05** | 6.7 (5.4), 6 (8.5) | | U = 644906; **p = 8.17e-03** | 9.13 (6.9), 8 (10) | U = 676118; **p = 1.40e-05** | |  |

*U = Mann-Whitney U test*. We applied a Bonferroni correction for 10 independent reasons for first using cannabis, setting the significance threshold at p<0.005. P-values below this threshold are marked with an asterisk.

## 2.9 Correlation between THC blood sample analytics with self-reported cannabis use measure.

The self-reported data included two measures: (1) frequency of use and (2) a THC unit estimate based on frequency and cannabis type, aligned with NIH guidelines (5 mg THC per unit). Analyses were conducted in the subsample

**Table S11. Exploring the THC blood sample levels and the self-reported information on cannabis use**

|  | **Variable** | **Statistics** | **Descriptor** |
| --- | --- | --- | --- |
| **Variables** | THC (Mean) ng/ml | M (SD), Median, % missing | 11.5 (10.8), 7.2, 14.8 |
|  | THC Unit | M (SD), Median, (IQR), % missing | 162 (306.6), 64 (205.5), 18.2 |
|  | Frequency | Never, N (%) | 10 (11.4) |
|  |  | Less than once or twice or past, N (%) | 8 (9.1) |
|  |  | Monthly or less, N (%) | 1 (1.1) |
|  |  | Weekly or less, N (%) | 5 (5.7) |
|  |  | More than once a week, N (%) | 11 (12.5) |
|  |  | Daily, N (%) | 52 (59.1) |
| **Correlation** | Between THC and THC unit | Correlation (r) Spearman, p-value | **0.77, p = 3.08e-13** |
| **Correlation** | Between THC and Frequency* | Correlation (r) Spearman, p-value | 0.63, p = 1.73e-09 |
| Polyserial correlation between Frequency and the THC mean (ng/ml) = 0.46 | | | |

M = mean; SD = Standard deviation; IQR = Interquartile Range, r = correlation, * Spearman correlation with frequency treated as continuous.

**Figure S7. Spearman correlation between THC blood concentration and weekly THC units**


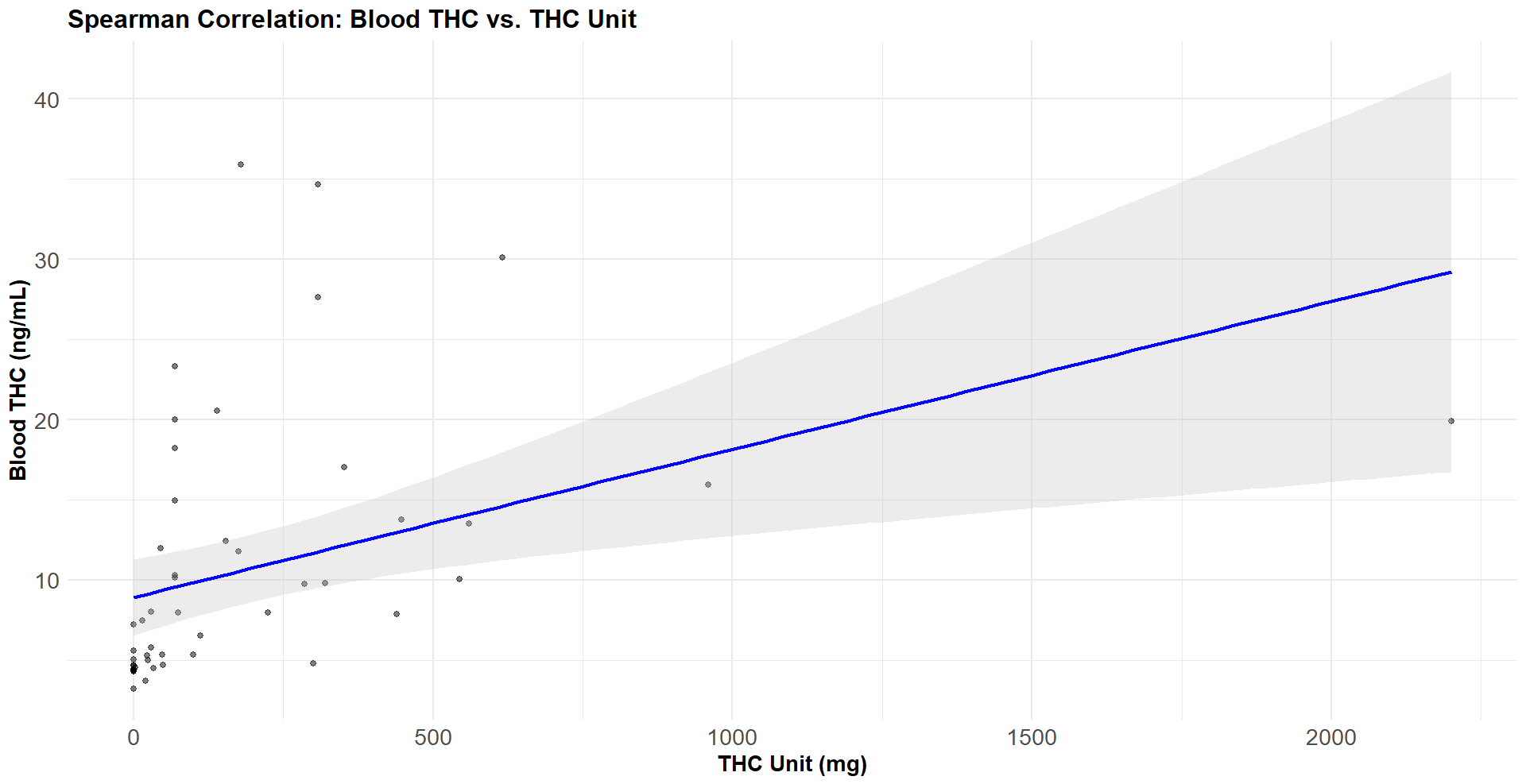


## 2.10 Sample representativeness

**Comparing the ethnic distribution of our CAMe online survey sample with the official 2021 ONS data**

**Figure S8. Raw numbers and percentages indicating the ethnic distribution in London according to the 2021 ONS estimates^7^**

**Figure S9. Raw numbers and percentages indicating the ethnic distribution in our CAMe online survey study**

## 2.11 Sensitivity analyses restricted to those who reported only a single RFUC

As previously reported, the CEQ (Cannabis Experiences Questionnaire) was built to allow participants to endorse multiple reasons for first using cannabis, and no attempt was made to prioritise or rank these responses. This approach was intentional to avoid the limitations of forced-choice formats and to capture better the multifaceted and overlapping motivations individuals may have. Accordingly, participants were included in all applicable RFUC categories within the regression models.

To address concerns about overlapping endorsement, we have added a series of sensitivity analyses restricted to participants who reported only a single RFUC. To ensure adequate statistical power, these analyses were limited to RFUC categories with at least 50 participants. RFUCs with lower sample sizes were excluded to reduce the risk of Type II error and unstable estimates. The results are reported below (Tables S12-S15).

**Table S12. The subsample of participants who reported only a single RFUC**

| **Variable** | **Count** | **Percentage** |
| --- | --- | --- |
| **Fun** | **94** | **9.25%** |
| **Friends** | **553** | **54.4%** |
| Family | 34 | 3.35% |
| **Curiosity** | **261** | **25.7%** |
| Boredom | 1 | 0.098% |
| Better—psychosis | 0 | NA |
| Better—physical discomfort | 8 | 0.79% |
| Better—pain | 21 | 2.07 |
| Better—depression | 22 | 2.17% |
| Better—anxiety | 22 | 2.17% |

In bold, RFUC ≥ 50 used for sensitivity analyses

**Table S13. *Linear regressions testing the association between RFUC and age at first cannabis use—sensitivity analyses restricted to participants who provided only a single RFUC***

|  |  |  |  |
| --- | --- | --- | --- |
|  | β (SE) | | p-value |
| **Friends** | -0.9 (0.24) | | **1.79e-04** |
| **Curiosity** | 0.23 (0.28) | | 0.4 |
| **Fun** | 0.38 (0.43) | | 0.37 |

β = beta coefficient; SE = Standard Error. *All the analyses were adjusted for sex, age, ethnicity, employment status, and years of education. We applied a Bonferroni correction for 10 independent reasons for first using cannabis, setting the significance threshold at p<0.005.

**Table S14. Adjusted linear regressions to test the associations between RFUC and the winsorised THC unit*—sensitivity analyses restricted to participants who provided only a single RFUC***

|  | **THC Unit** | | **THC Unit – IMPUTED MODEL** | | |  |
| --- | --- | --- | --- | --- | --- | --- |
|  | β (SE) | p-value | β (SE) | | p-value | |
| **Friends** | -28.3 (15.9) | 0.07 | -41.6 (12.8) | | **1.24e-03** | |
| **Curiosity** | 7.9 (17.7) | 0.66 | 18.5 (14.6) | | 0.2 | |
| **Fun** | -14.7 (26.6) | 0.6 | -7.3 (22.1) |  | 0.7 | |

β = beta coefficient; SE = Standard Error. *All the analyses were adjusted for sex, age, ethnicity, employment status, and years of education. In all our analyses, we applied a winsorised version of the THC unit. We applied a Bonferroni correction for 10 independent reasons for first using cannabis, setting the significance threshold at p<0.005.

**Table S15. Adjusted linear regressions to test the associations between RFUC and 1) GPTS-tot, 2) GAD-7, and 3) PHQ-9*—sensitivity analyses restricted to participants who provided only a single RFUC***

|  | **GPTS-TOTAL** | | **GAD** | | **PHQ-9** | | |
| --- | --- | --- | --- | --- | --- | --- | --- |
|  | β (SE) | p-value | β (SE) | p-value | β (SE) |  | p-value |
| **Friends**  **Adjusted for THC unit** | 1.2 (1.41)  1.46 (1.69) | 0.39  0.39 | -0.41 (0.34)  -0.34 (0.4) | 0.22  0.39 | -0.31 (0.39)  -0.21 (0.47) | | 0.43  0.65 |
| **Curiosity**  **Adjusted for THC unit** | -2.33 (1.59)  -2 (1.87) | 0.14  0.28 | 0.18 (0.38)  0.15 (0.44) | 0.64  0.73 | 0.27 (0.45)  0.26 (0.52) | | 0.55  0.62 |
| **Fun**  **Adjusted for THC unit** | -3.2 (2.4)  -2.1 (2.82) | 0.19  0.28 | -0.63 (0.59)  -0.43 (0.67) | 0.28  0.52 | -0.55 (0.68)  -0.35 (0.79) | | 0.42  0.66 |

β = beta coefficient; SE = Standard Error. All the analyses were adjusted for sex, age, ethnicity, employment status, and years of education. Additional adjustment for THC is also reported. We applied a Bonferroni correction for 10 independent reasons for first using cannabis, setting the significance threshold at p<0.005.

## 2.12 Full version of Table 2 reporting the full statistics

**Table S16. Adjusted linear regressions to test the associations between RFUC and 1) GPTS-tot, 2) GAD-7, and 3) PHQ-9**

|  | **GPTS-TOTAL** | | | **GAD** | | | **PHQ-9** | | |
| --- | --- | --- | --- | --- | --- | --- | --- | --- | --- |
|  | β (SE) | 95% CI | p-value | β (SE) | 95% CI | p-value | β (SE) | 95% CI | p-value |
| **Friends**  **Adjusted for THC unit** | -0.33 (0.83)  0.25 (0.96) | -1.96, 1.3  -1.63, 2.13 | 0.69  0.79 | -0.24 (0.2)  -0.17 (0.23) | -0.63, 0.15  -0.62, 0.28 | 0.23  0.45 | -0.02 (0.24)  0.05 (0.28) | -049, 0.45  -0.5, 0.6 | 0.93  0.85 |
| **Family**  **Adjusted for THC unit** | 3.16 (1.24)  2.15 (1.41) | 0.73, 5.6  -0.6, 4.9 | 1.1e-02  **5e-03** | 0.76 (0.3)  0.56 (0.3) | 0.17, 1.35  -0.03, 1.15 | 1.02e-02  9e-02 | 1.11 (0.36)  0.82 (0.41) | 0.4, 1.82  0.02, 1.63 | **1.88e-03**  0.04 |
| **Better—physical discomfort**  **Adjusted for THC unit** | 8.63 (1.59)  8.89 (1.75) | 5.51, 11.75  5.46, 12.32 | **6.51e-08**  **4.12e-07** | 0.99 (0.38)  1.07 (0.41) | 0.25, 1.73  0.27, 1.87 | 8.43e-03  **0.01** | 1.29 (0.46)  1.42 (0.51) | 0.39, 2.19  0.42, 2.42 | **5.04e-03**  5.03e-02 |
| **Better—pain**  **Adjusted for THC unit** | 8.14 (1.44)  7.24 (1.59) | 5.32, 10.96  4.12, 10.36 | **1.63e-08**  **5.56e-06** | 1.41 (0.34)  1.31 (0.38) | 0.74, 2.08  0.57, 2.05 | **3.94e-05**  **4.75e-04** | 1.82 (0.41)  1.57 (0.46) | 1.02, 2.62  0.67, 2.47 | **1.07e-05**  **6.41e-04** |
| **Better—anxiety**  **Adjusted for THC unit** | 9.56 (1.04)  9.67 (1.16) | 7.52, 11.6  7.4, 11.94 | **8.1e-20**  **1.63e-16** | 2.69 (0.25)  2.66 (0.27) | 2.2, 3.18  2.13, 3.19 | **2.42e-27**  **4.35e-22** | 3.2 (0.3)  3.07 (0.3) | 2.61, 3.79  2.48, 3.66 | **1.56e-26**  **7e-20** |
| **Better—depression**  **Adjusted for THC unit** | 9.7 (1.10)  9.12 (1.22) | 7.54, 11.86  6.73, 11.51 | **1.6e-18**  **1.21e-13** | 2.38 (0.26)  2.28 (0.29) | 1.87, 2.89  1.71, 2.85 | **9.11e-20**  **3.65e-15** | 3.72 (0.31)  3.60 (0.35) | 3.11, 4.33  2.91, 4.29 | **4.25e-32**  **1.38e-24** |
| **Better—psychosis**  **Adjusted for THC unit** | 20.89 (3.9)  16.5 (4.26) | 13.25, 28.5  8.15, 24.85 | **9.14e-08**  **0.0001** | 4.35 (0.93)  4.02 (1) | 2.53, 6.17  2.06, 5.98 | **2.68e-06**  **6.26e-05** | 5.26 (1.12)  5.12 (1.22) | 3.06, 7.46  2.73, 7.51 | **2.74e-06**  **3.06e-05** |
| **Curiosity**  **Adjusted for THC unit** | -3.49 (0.79)  -2.61 (0.93) | -5.03, -1.94  -4.43, -0.79 | **9.32e-06**  **0.005** | -0.47 (0.19)  -0.44 (0.22) | -0.8, -0.1  -0.87, -0.01 | 0.01  4.18e-02 | -0.23 (0.23)  -0.19 (0.27) | -0.68, 0.22  -0.72, 0.34 | 0.3  0.48 |
| **Fun**  **Adjusted for THC unit** | -3.88 (0.76)  -3.71 (0.9) | -5.37, -2.4  -5.74, -1.95 | **3.39e-07**  **3.49e-05** | -0.73 (0.18)  -0.73 (0.21) | -1.08, -0.38  -1.14, -0.32 | **5.39e-05**  **6.04e-04** | -0.55 (0.22)  -0.55 (0.26) | -0.98, -0.12  -1.1, -0.04 | 1.23e-02  3.29e-02 |
| **Boredom**  **Adjusted for THC unit** | 3.66 (1.17)  3.06 (1.31) | 1.37, 5.95  0.49, 5.63 | **1.79e-03**  0.019 | 0.65 (0.28)  0.52 (0.31) | 0.1, 1.2  -0.09, 1.13 | 1.97e-02  9.12e-02 | 1.34 (0.34)  1.09 (0.38) | 0.67, 2  0.35, 1.83 | **6.96e-05**  **3.8e-03** |

β = beta coefficient; SE = Standard Error. All the analyses were adjusted for sex, age, ethnicity, employment status, and years of education. Additional adjustment for THC is also reported. We applied a Bonferroni correction for 10 independent reasons for first using cannabis, setting the significance threshold at p<0.005.

**REFERENCES**

1. Di Forti M, Quattrone D, Freeman TP, et al. The contribution of cannabis use to variation in the incidence of psychotic disorder across Europe (EU-GEI): a multicentre case-control study. *Lancet Psychiatry*. May 2019;6(5):427-436. doi:10.1016/S2215-0366(19)30048-3

2. Cousijn J, Kuhns L, Filbey F, Freeman TP, Kroon E. Cannabis research in context: The case for measuring and embracing regional similarities and differences. *Addiction*. Sep 2024;119(9):1502-1504. doi:10.1111/add.16460

3. Potter DJ, Hammond K, Tuffnell S, Walker C, Di Forti M. Potency of Delta(9) -tetrahydrocannabinol and other cannabinoids in cannabis in England in 2016: Implications for public health and pharmacology. *Drug Test Anal*. Apr 2018;10(4):628-635. doi:10.1002/dta.2368

4. Office for National Statistics (ONS). Crime in England and Wales, year ending June 2022 (2022).

5. Spinazzola E, Quattrone D, Rodriguez V, et al. The association between reasons for first using cannabis, later pattern of use, and risk of first-episode psychosis: the EU-GEI case-control study. *Psychol Med*. Nov 2023;53(15):7418-7427. doi:10.1017/S0033291723001071

6. Freeman T, Lorenzetti V. Moving forwards with the standard THC unit *Addiction*. 2020;115(7):1222-1223. doi:10.1111/add.15107

7. Office for National Statistics (ONS). Ethnic distribution in Greater London: Census 2021 data from Nomis. <https://www.nomisweb.co.uk> January 17, 2025.
